# Spatially convergent functional MRI signatures of diabetes and male sex identify genetic vulnerabilities to accelerated brain ageing

**DOI:** 10.1093/braincomms/fcag347

**Published:** 2026-09-19

**Authors:** Anthony G Chesebro, Luna R Rohm, Botond B Antal, Corey Weistuch, Helmut H Strey, Eva-Maria Ratai, David T Jones, Lilianne R Mujica-Parodi

**Affiliations:** Laufer Center for Physical and Quantitative Biology, State University of New York at Stony Brook, Stony Brook, NY 11794, USA; Athinoula A. Martinos Center for Biomedical Imaging, Massachusetts General Hospital and Harvard Medical School, Boston, MA 02129, USA; Athinoula A. Martinos Center for Biomedical Imaging, Massachusetts General Hospital and Harvard Medical School, Boston, MA 02129, USA; Universitätsmedizin Charité, Berlin 10117, Germany; Laufer Center for Physical and Quantitative Biology, State University of New York at Stony Brook, Stony Brook, NY 11794, USA; Athinoula A. Martinos Center for Biomedical Imaging, Massachusetts General Hospital and Harvard Medical School, Boston, MA 02129, USA; Department of Medical Physics, Memorial Sloan Kettering Cancer Center, New York, NY 10065, USA; Laufer Center for Physical and Quantitative Biology, State University of New York at Stony Brook, Stony Brook, NY 11794, USA; Athinoula A. Martinos Center for Biomedical Imaging, Massachusetts General Hospital and Harvard Medical School, Boston, MA 02129, USA; Athinoula A. Martinos Center for Biomedical Imaging, Massachusetts General Hospital and Harvard Medical School, Boston, MA 02129, USA; Department of Neurology, Mayo Clinic, Rochester, MN 55905, USA; Laufer Center for Physical and Quantitative Biology, State University of New York at Stony Brook, Stony Brook, NY 11794, USA; Athinoula A. Martinos Center for Biomedical Imaging, Massachusetts General Hospital and Harvard Medical School, Boston, MA 02129, USA; Santa Fe Institute, Santa Fe, NM 87501, USA

**Keywords:** ageing, functional neuroimaging, type 2 diabetes, male sex, spatial transcriptomics

## Abstract

Age-related cognitive decline results from complex interactions between neuroendocrine and neurometabolic processes that undergo lifelong degradation, yet the mechanisms underlying these interactions remain poorly understood. This study examined the effects of diabetes and sex on functional brain networks across ageing through analysis of two large cohorts (*N* = 1621 total) using both 3T and 7T functional MRI, complemented by spatial transcriptomic data from over 14 000 genes from six post-mortem brains. Four networks—cingulo-opercular, default mode, salience and lateral somatomotor—exhibited significant functional decline in both individuals with diabetes and independently in males. Gene expression analysis of vulnerable networks revealed significant over-expression of insulin-dependent glucose transporters, dopaminergic and GABAergic synaptic genes and VEGFA–VEGFR2 pathway components. These findings suggest functionally-specific circuit vulnerability to metabolic and hormonal dysregulation, potentially offering targets for early intervention before irreversible neurodegeneration occurs.

## Teaser

Chesebro *et al*. demonstrate that specific brain networks, defined by functional MRI, experience accelerated ageing in both individuals with diabetes and males. Spatial transcriptomics show these vulnerable networks overexpress insulin-dependent glucose transporters, dopaminergic and GABAergic synaptic genes and vascular signaling pathway components, suggesting functionally-specific circuit vulnerability to metabolic and hormonal dysregulation.

## Graphical Abstract

**Graphical Abstract:**
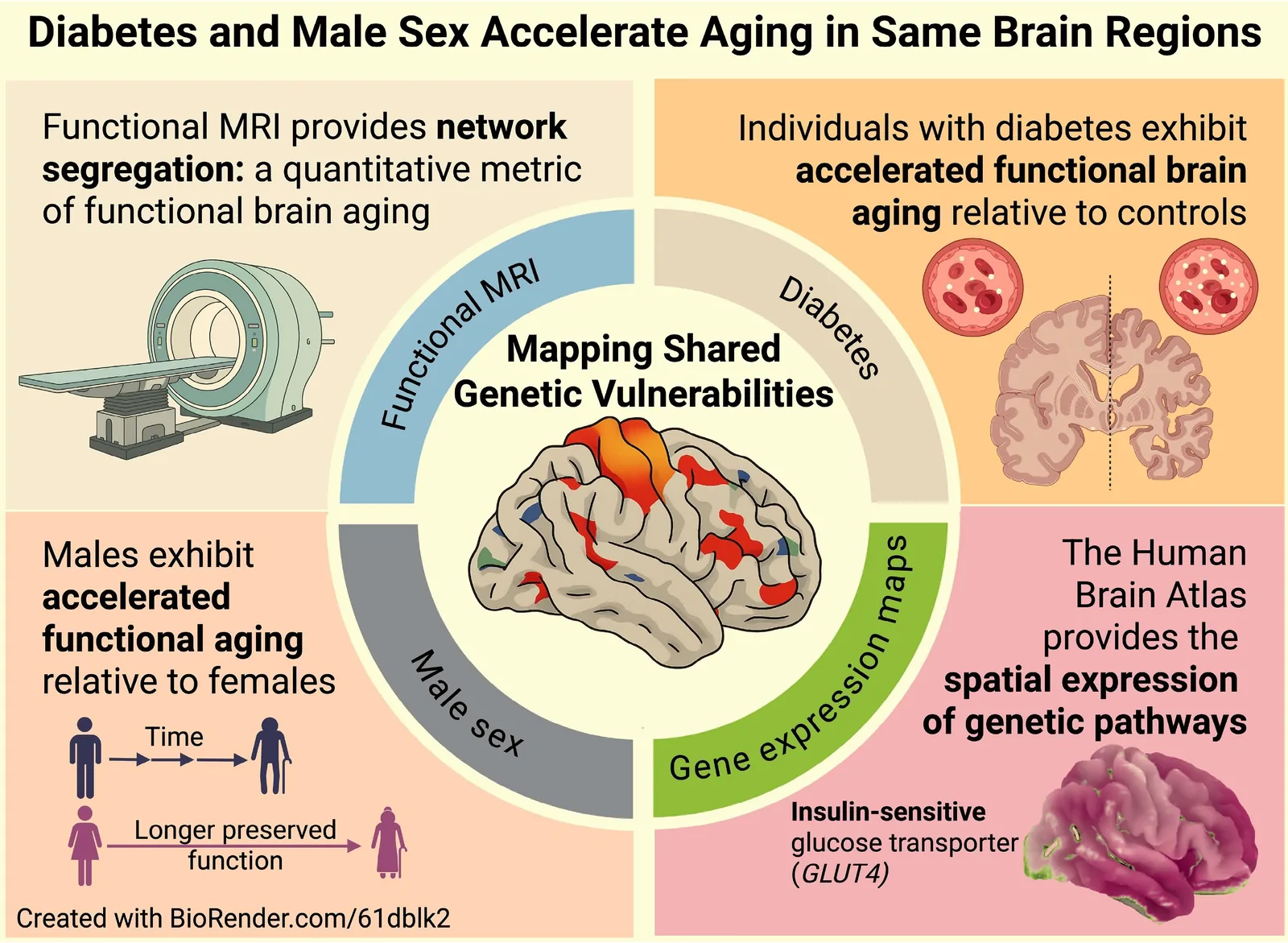

## Introduction

The maintenance of brain homeostasis in the face of extreme variability experienced over several decades of life requires neuroendocrine and neurometabolic coordination.^1-3^ Due to this inherently multi-system feedback, age-related cognitive impairment and dementia are often symptomatic results of a diverse constellation of underlying pathological processes that often co-occur in ways that challenge diagnosis and prevention.^4,5^ Final assessments of disease burden rely on measures of neurodegeneration, often informed by grey^6^ and white matter^7^ structural abnormalities visible with MRI, or by other imaging biomarkers of corresponding pathologies, such as tau-PET. These changes are typically diagnostic at the end stage of disease when functional decline is already clinically apparent.^4^ In the context of dementia and Alzheimer’s disease, functional decline is observed much earlier on either functional MRI (fMRI) or metabolic imaging (FDG-PET) than most other biomarkers, often co-occurring with early biomarkers of disease accumulation.^8,9^ Examining the effects of neurometabolic and neuroendocrine conditions on functional metrics is therefore an appealing prospect, as functional changes observed in ageing often are sensitive to earlier disease processes and can provide insights before cognitive and behavioral impairment becomes apparent.^9^

Disrupted energy homeostasis is a commonly implicated mechanism within the described constellation of pathological processes.^2,10-13^ The brain maintains a delicate balance between the dominance of interregional connections for preserving functional circuits and the metabolic costs associated with maintaining these circuits.^10,14,15^ The brain is therefore uniquely vulnerable to disorders that impact energy homeostasis.^1,13,16^ Diabetes, as both an endocrine and metabolic disorder, has been increasingly implicated as detrimental in brain ageing, with Alzheimer’s disease being termed in some works as ‘Type 3 diabetes’ as the effects of disordered metabolic processes feature so significantly in age-related cognitive impairment.^10,17-19^ Type 2 diabetes mellitus, in particular, has been robustly associated with region-specific neurological insult and subsequent cognitive impairment.^10,20^ As the global burden of diabetes continues to grow,^21^ understanding and mitigating the effects of this chronic degenerative disease on brain health is a high priority for public health.

Sex hormones over the lifespan also lead to striking differences between cognitive ageing in males versus females,^22,23^ often in ways that interact with other neuroendocrine conditions like diabetes.^19^ There is some evidence of an estrogen-protective effect on cognitive ageing,^24^ with longer exposure to estrogen associated with preserved cognitive function in females,^25,26^ albeit with a potentially steeper eventual decline in late age.^22^ This functionally protective effect occurs even though females typically have higher degrees of pathology associated with Alzheimer’s disease and vascular dementia.^27^ This suggests that the relative functional benefits experienced by females compared to males during ageing may not necessarily be due to mitigation of underlying pathology but to different functional alterations in the face of these pathologies, such as different compensatory vascular^28^ and metabolic^29^ mechanisms.

**Figure 1:**
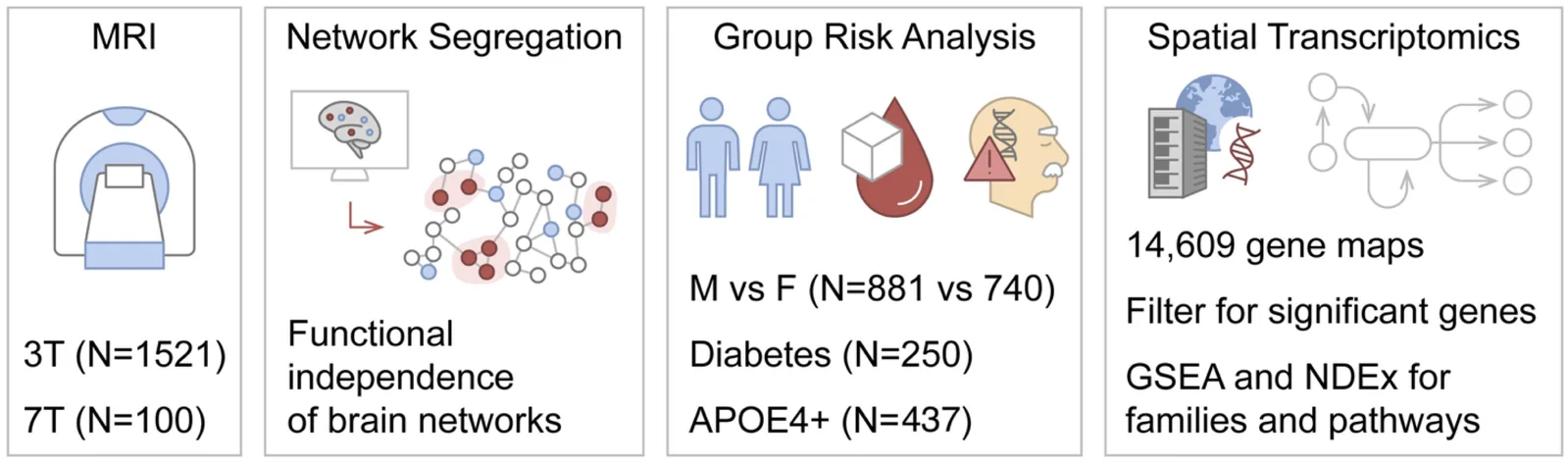
**Schematic representation of study workflow.** Two cohorts received resting-state functional MRI scans at 3T (single day) or 7T (2 days, repeated scans). Both cohorts were processed to extract functional network segregation values, as this is a biomarker of age-related functional decline. We then analyzed these groups to identify spatially localized deficits in three subgroups: males compared to females, participants with and without type 2 diabetes mellitus, and APOE4+ compared to APOE4− participants. Finally, to provide mechanistic insight to complement the functional findings, we used the Allen Human Brain Atlas^31^ to identify genes and the associated functional pathways that are relatively over- and under-expressed in the networks that showed co-susceptibility to diabetes and male sex-related functional decline.

Here, we aim to identify the relationship between these effects by examining two large cohorts (over 1600 individuals in total) that received extensive clinical and fMRI evaluations at both high (3T) and ultra-high (7T) fields using the analysis outlined in Fig. 1. First, we demonstrate that functional network segregation, known to be associated with both age- and metabolic-related changes in the brain,^15,30^ exhibited accelerated decline in specific regions in both participants with diabetes and in males. We demonstrate that these effects are spatially congruent but are in fact independent effects not driven by baseline differences in metabolism between males and females. We also demonstrate that these effects are spatially and statistically independent from APOE4 status. Taking the regions identified as vulnerable to accelerated functional decline in both diabetes and males, we then use spatial transcriptomic data from over 14 000 genes from a post-mortem dataset^31^ to identify candidate genes and their related pathways that could give rise to these co-localized deficits.

## Materials and methods

### Participants and fMRI acquisition—3T dataset

The participant cohort for the 3T fMRI dataset is composed of data from the Mayo Clinic Study of Aging, which has previously been described in detail.^32^ The subset of data analyzed in this study contained data for 1521 participants between 31 and 90 years old. Complete clinical data acquired, including diabetes status, fasting blood glucose, HbA1c levels, cognitive impairment and APOE4 status, is reported in Table 1. Additional details of participant recruitment and clinical data acquisition are included in Roberts *et al.*,^32^ with additional notes about the specific data included in this study in the Supplementary Methods as well. Structural MRI and fMRI scans in this dataset were acquired on a 3T GE Discovery MR750. The imaging protocol included an echo-planar imaging sequence for whole-brain blood oxygen level-dependent (BOLD) signals and a T1-weighted imaging sequence for structural images. BOLD acquisition parameters comprised a repetition time of 2.9 s, echo time (TE) of 30 ms, flip angle of 90°, voxel size of 3.28 mm × 3.28 mm × 3.3 mm and total acquisition length of 7 min 44 s (160 volumes). T1-weighted structural volumes were acquired with 1.2 mm × 1.02 mm × 1.02 mm voxel size, TE = 3.044 ms, repetition time = 7.36 ms and flip angle of 8.0°.

**Table 1:** Descriptive statistics of both cohorts

	3T dataset (*N* = 1521)			7T dataset (*N* = 100)		
	Male	Female	Statistic	Male	Female	Statistic
Sex, *N*	829	692	—	52	48	—
Age, mean (SD)	70 (12)	69 (12)	*t* = −2.0, *P* = 0.04	45 (17)	45 (16)	*t* = 0.20, *P* = 0.84
Diabetes (T1 or T2), *N* (%)	160 (19)	90 (13)	***χ*** ^2^ = 11, *P* < 0.01	0 (0)	0 (0)	—
Fasting blood glucose, mean (SD)	102 (29)	97 (22)	*t* = −2.6, *P* < 0.01	85 (8.5)	82 (8.5)	*t* = −1.8, *P* = 0.08
HbA1c	5.67 (0.78)	5.59 (0.66)	*t* = −1.2, *P* = 0.22	5.17 (0.29)	5.17 (0.33)	*t* = −0.07, *P* = 0.94
Cognitive impairment, *N* (%)	101 (12)	56 (8)	***χ*** ^2^ = 6.8, *P* < 0.01	0 (0)	0 (0)	—
APOE4 positive, *N* (%)	223 (27)	214 (31)	***χ*** ^2^ = 3.0, *P* = 0.08	—	—	—

### Participants and fMRI acquisition—7T dataset

A second, independent fMRI dataset was used to evaluate the replicability of the observed age and sex-specific associations with functional network segregation. This dataset was acquired at 7T field strength from a cohort of *N* = 100 healthy adults aged between 21 and 79 years. This study was conducted under the oversight of the Internal Review Board at Massachusetts General Hospital (Boston, MA, USA) and State University of New York at Stony Brook (Stony Brook, NY, USA). Informed consent was obtained prior to a physical examination and administration of cognitive testing to ensure cognitive health (see Supplementary Methods for details). Each participant was scanned on two separate occasions, at least 24 h apart, using identical procedures.

Structural MRI and fMRI scans in this dataset were acquired on a 7T Siemens Magnetom (Siemens Healthineers, Erlangen, Germany). Similar to the 3T dataset, the imaging protocol echo-planar imaging-BOLD fMRI and T1-weighted structural sequences. BOLD images were captured using a protocol for detecting resting-state networks, with acquisition parameters optimized via a dynamic phantom (BrainDancer; ALA Scientific Instruments).^33^ The resulting protocol for BOLD acquisition included a simultaneous multi-slice slice acceleration factor of 5, *R* = 2 acceleration in the primary phase encoding direction (48 reference lines) and online generalized autocalibrating partially parallel acquisition image reconstruction. Additional acquisition parameters comprised a repetition time of 802 ms, TE of 20 ms, flip angle of 33°, voxel size of 2 mm × 2 mm × 1.5 mm and total acquisition length of 10 min (750 volumes). T1-weighted structural volumes were acquired with 1-mm isotropic voxel size and four echoes using a protocol with TE1 = 1.61 ms, TE2 = 3.47 ms, TE3 = 5.33 ms, TE4 = 7.19 ms, repetition time = 2530 ms, flip angle of 7.0°, *R* = 2 acceleration in the primary phase encoding direction (32 reference lines) and online generalized autocalibrating partially parallel acquisition image reconstruction.

### fMRI preprocessing and network segregation

The fMRI data from both the 3T and 7T datasets were preprocessed using a combination of methods from *fMRIprep*,^34^ SPM (SPM12, UCL) and the *nilearn* Python library^35^ as described in previous work.^12^ The T1-weighted anatomical images first underwent bias field correction, skull-stripping and normalization to Montreal Neurological Institute templates. Functional BOLD contrast images were then realigned to correct for head motion, slice-time corrected and adjusted for geometric distortions caused by magnetic field inhomogeneities by registering the BOLD reference to the intensity-inverted T1-reference.^36^ Following this, the functional images were coregistered with the anatomical scans and normalized to Montreal Neurological Institute space. To minimize physiological noise, the time-series were bandpass filtered between 0.01 and 0.1 Hz, and mean signals from white matter and cerebrospinal fluid voxels were regressed out. Additionally, six motion parameters were also regressed out reduce the effect of motion-related artifacts. Spatial smoothing was applied to the data using a Gaussian kernel with a full width at half maximum of 5 mm. Finally, the time-series were parcellated into 300 functional regions of interest (ROIs) using the Seitzman atlas for subsequent analyses.^37^

Network segregation was computed as the difference of within network and between network connectivity, given as previously defined in Manza *et al.*^15^ by


$$\frac{{\bar{Z}}_{W}-{\bar{Z}}_{B}}{{\bar{Z}}_{W}},$$

where ${\bar{Z}}_{W}$ and ${\bar{Z}}_{B}$ are the average Fisher-transformed correlation for nodes within and between networks, respectively. It is important to note that this average is of positive correlations only and that all positive correlations are used in the average, regardless of weight. Only positive correlations are included because: (1) the physiological relevance of negative correlations in fMRI functional connectivity analyses is disputed^38-40^; (2) including negative correlations would deviate from the standard definition of network segregation used in prior literature and thus limit interpretation in conversation with these works^15,40^; and (3) the original work defining network segregation showed no meaningful change in results if negative weights were excluded.^30^

### Statistical methods

**Figure 2:**
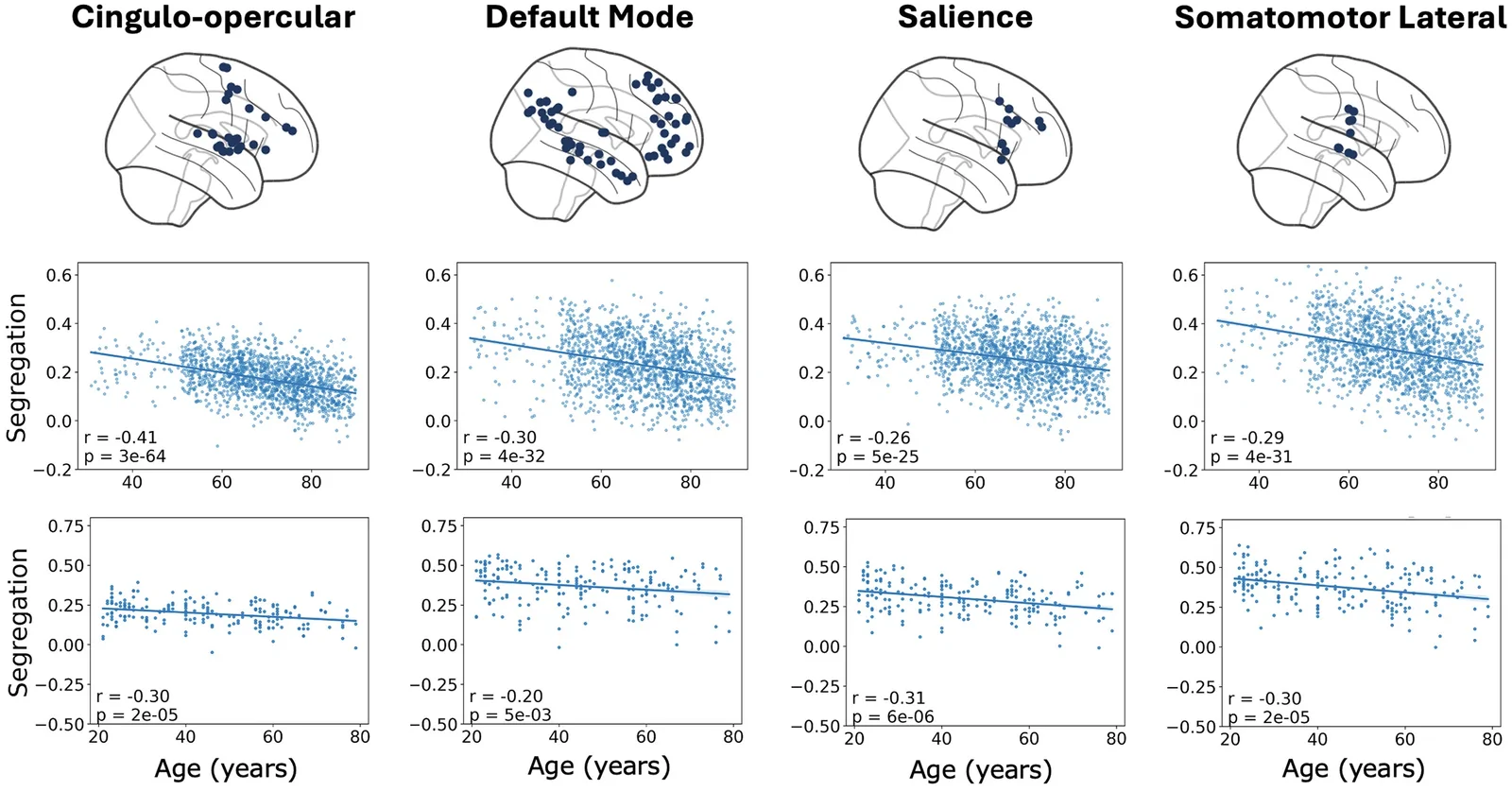
**Loss of functional network segregation is associated with age throughout the brain.** The first row shows the ROI locations for the functional networks of interest (cingulo-opercular, default mode, salience and somatomotor lateral networks). Full 3D locations to visualize these coordinates are presented in Supplementary Fig. 1. (**A**) 3T fMRI dataset results show network segregations show a strong decrease correlated with increasing age. This analysis repeated across all networks is illustrated in Supplementary Fig. 2. (**B**) 7T fMRI dataset results show similarly strong associations of decreased network segregation with age. This analysis repeated across all networks is illustrated in Supplementary Fig. 3. Lines represent linear regression between segregation and age with 68th percentile bootstrapped confidence interval shaded.

**Figure 3:**
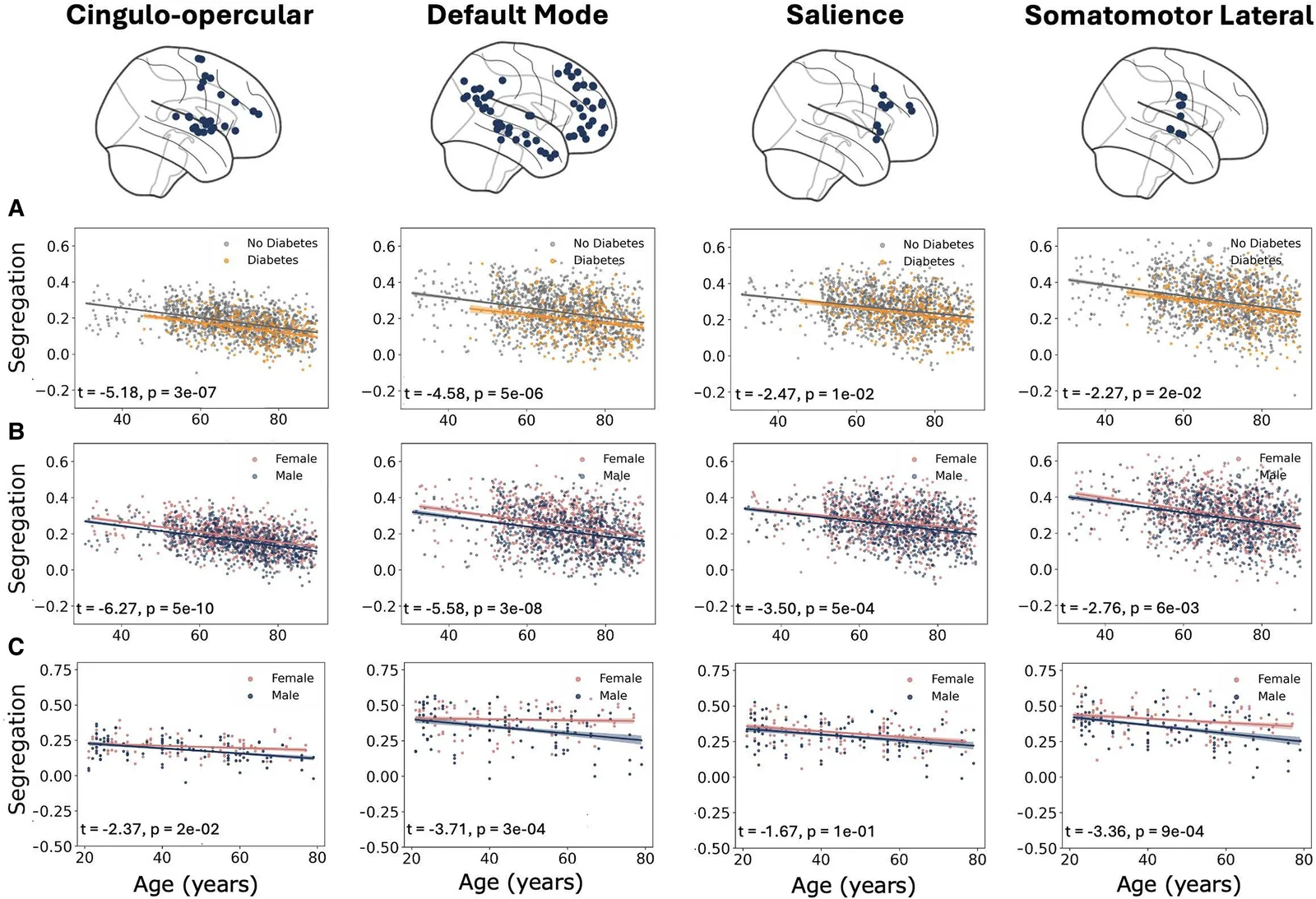
**Spatial overlap between effects of diabetes and male sex on accelerated decrease of network segregation with age.** Four specific networks show decreased network segregation in diabetes compared to controls as well as in males compared to females in both datasets. (**A**) Diabetes significantly decreases network segregation at all age points in the illustrated networks. (**B**) Male sex is associated with decreased network segregation in the same networks at all ages in the 3T dataset. (**C**) Male sex is associated with decreased network segregation in the 7T replication dataset as well. In all plots, lines represent linear regression between segregation and age with 68th percentile bootstrapped confidence interval shaded. Statistics are differences between groups (diabetes versus no diabetes in **A**, male versus female in **B** and **C**), corrected for age.

**Figure 4:**
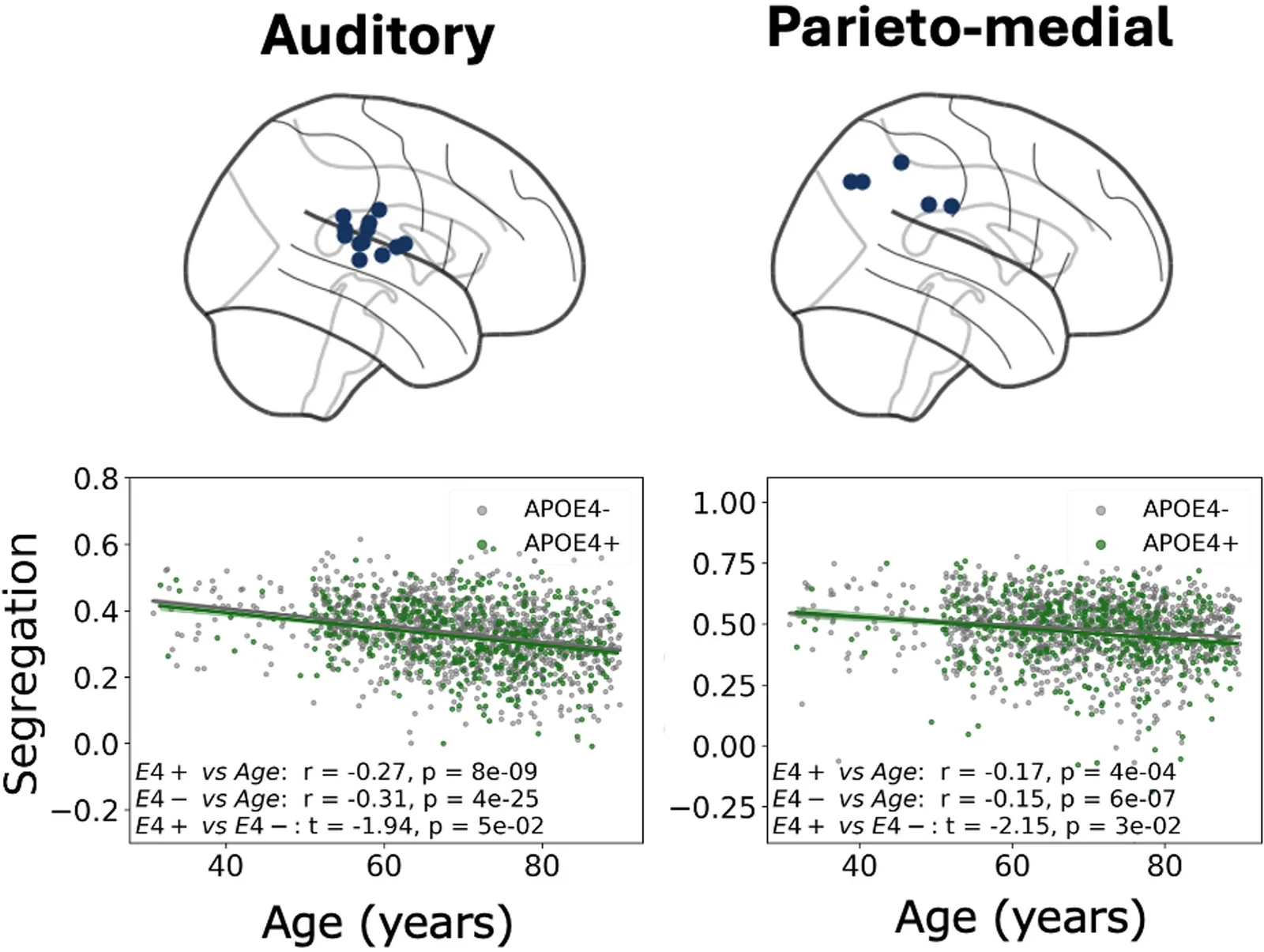
**APOE4 positive status is associated with auditory and parieto-medial network functional segregation decrease.** This is a notably spatially distinct pattern from the diabetes and sex effects, suggesting that they are independent pathways in early functional decline. Lines represent linear regression between segregation and age with 68th percentile bootstrapped confidence interval shaded.

Associations with age as presented in Fig. 2 are simple Pearson correlations with linear regressions and the standard error of the regression visualized as well. For all comparisons of variables of interest (diabetes, sex, APOE4 status and repeated scans) presented in Figs 3 and 4, Supplementary Figs 2–8 and Supplementary Tables 1–6, ordinary least squares (OLS) regression for multiple independent variables or random effects were employed as appropriate. All models that assessed interaction effects included main effects of both variables as well. Additional statistical methods to assess significance of spatial overlap with gene maps are discussed further below, and additional quality check statistics for the fMRI and gene set enrichment analysis (GSEA) pipelines are discussed further in the Supplementary Methods and illustrated in Supplementary Figs 9–11.

### Gene expression analyses

Regional microarray expression data were obtained from six post-mortem brains (one female, 24–57 years old, 42.5 ± 3.4) provided by the Allen Human Brain Atlas^31^ (https://human.brain-map.org). Data were processed with the *abagen* toolbox (version 0.1.3; https://github.com/rmarkello/abagen) using a 300-region functionally defined volumetric atlas in Montreal Neurological Institute space.^37^ Our analysis pipeline followed the standard *abagen* pipeline with minor variations as detailed in Supplementary Methods. This process resulted in expression maps for 16 826 genes throughout the brain. These resulting expression maps were then filtered for genes included in brain-specific gene sets,^41^ selecting 87% (14 609) of the gene maps for subsequent analyses.

To determine which genes were most associated with the functional regions most affected by diabetes and male sex, the average expression levels were computed for these ROIs (cingulo-opercular, default mode, salience and somatomotor lateral networks) and then used to calculate the ratio against the expression levels for the remaining ROIs to compute the relative over/under-expression of the genes in these ROIs compared with the rest of the brain. To correct for potential spatial autocorrelations within the gene maps, the toolbox *neuromaps*^42^ was used to create null spatial maps exhibiting similar autocorrelation patterns.^43^ For each gene selected, 1000 null maps were produced and the ratio between affected and unaffected regions were computed in the same way as the true ratios, yielding a distribution of expression ratios across all permutations. The resulting ratios were combined, and a gamma distribution was fit to them, allowing for the quantification of statistical significance between the observed gene expression ratio and the potential distribution from null maps. This allowed for an unbiased, direct quantification of the significance of a correlation between a functional spatial map and a gene of interest while controlling for spatial autocorrelation of genetic samples. To address multiple comparisons, the *P*-values obtained from this fit were false-discovery rate (FDR)-corrected.^44^ An initial analysis was done using the same method for the APOE-associated functional networks (both parieto-medial alone and parieto-medial and auditory networks combined). As this analysis yielded extremely weak associations with all genes, it is excluded from the results.

Using the ratios and statistical significance values calculated from the above steps, two analyses were then performed: a targeted analysis of genes hypothesized *a priori* to be potentially related to pathways of interest, along with control genes that should show no significant correlations with the contrasts of interest; and an unsupervised analysis consisting of taking the genes most significantly associated with the spatial patterns observed in the functional results and analyzing which pathways these belonged to. For the targeted analysis, genes hypothesized to be associated with diabetes (e.g. *SLC2A4* and *SLC2A12*) and sex-specific processes (e.g. *ESR1* and *GRM1*) were selected and compared to control genes that should have no spatial relationship with the effects of interest (e.g. insulin-insensitive glucose transporters, inhibin receptors). For the unsupervised analyses, the top 500 most statistically significant genes (both over and under-expressed in the diabetes and sex-specific effect networks) were used in a GSEA (500 genes being the maximum cutoff allowed by the tool). Standard GSEA software (https://www.gsea-msigdb.org^45^) was used for this analysis, specifically focused on C5 (ontology gene sets) overlap from the molecular signatures database (MSigDB^46^). This unsupervised approach was extended using the same 500 genes as a query for related pathways using the Network Data Exchange (NDEx^47^).

**Figure 5:**
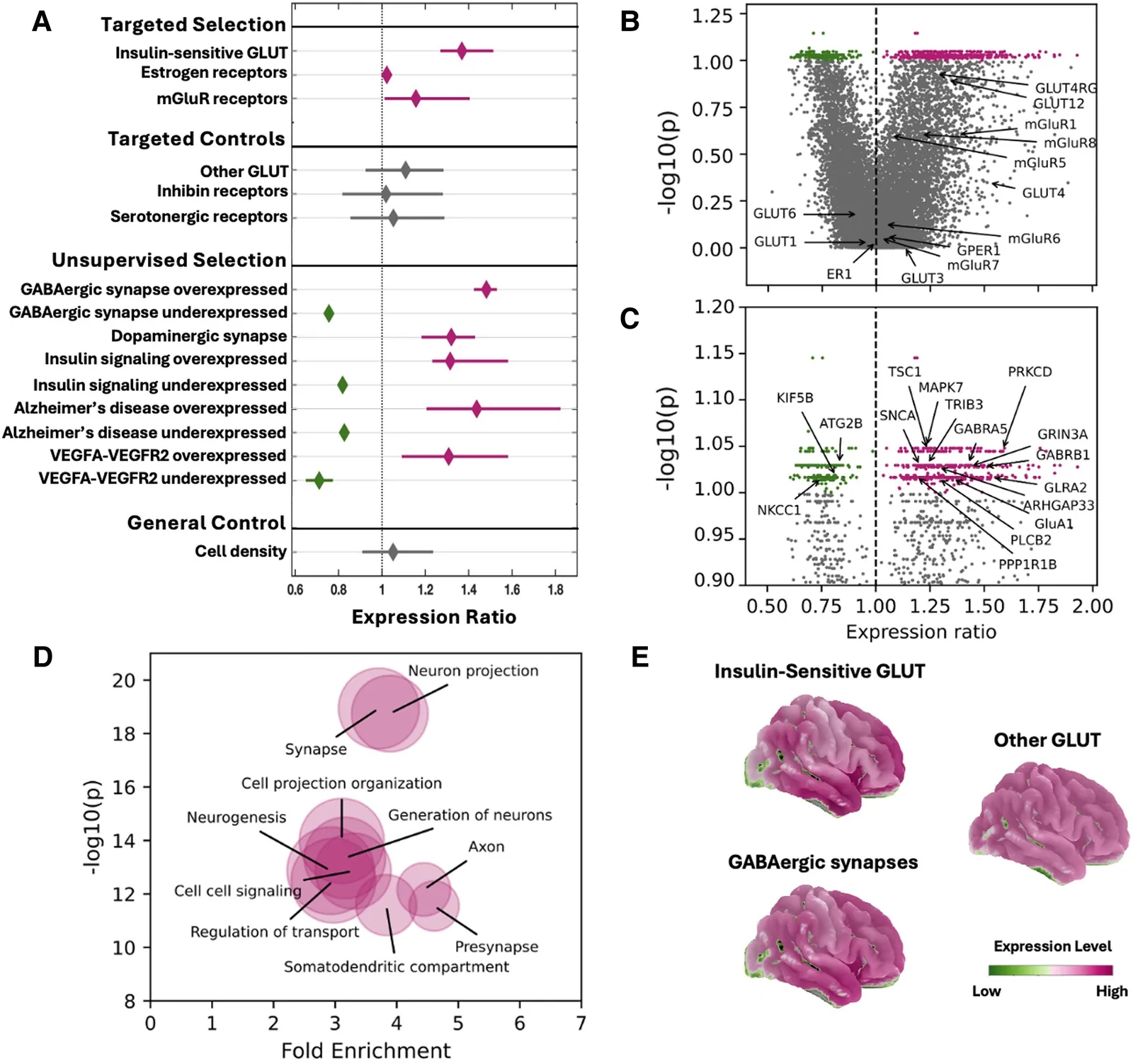
**Spatial transcriptomics implicate insulin signaling, synaptic activity and vascular signaling in region-specific functional decline.** (**A**) Relative expression levels of the different genes selected for targeted hypotheses and controls, as well as those shown to have significantly higher expression by random selection. Full gene lists for each category can be found in Supplementary Table 7. Expression ratio is the relative level of mRNA expression in the four networks of interest compared to the rest of the brain. Bars are drawn showing the range of expression ratios of the gene set (bounds are the minimum and maximum ratios), with diamond centers at the mean expression ratio of all selected genes in the set. Full description of expression ratios and null map generation provided in the statistical methods. (**B**) Entire set of relative expression levels for all genes, with *P*-values computed based on spatially-correlated null surrogate maps.^43^ Each point represents the ratio of mRNA expression of that gene in the four networks of interest compared to the rest of the brain. Specific genes from the targeted hypotheses are shown with arrows, with selected genes typically having expression ratios well above 1, while control genes are relatively uniform throughout the brain. Full description of expression ratios, null map generation and bootstrapped significance testing provided in the statistical methods. (**C**) Zoomed section of the upper portion of **B**, with specific genes from the unsupervised selection highlighted. (**D**) GSEA results showing the top 10 associated gene set collections in the full, unsupervised analysis, implicating a predominance of neuronal function and extracellular signaling pathways. This is expanded in Supplementary Fig. 9 to different number of gene cutoffs used in the GSEA. (**E**) Example spatial distributions of specific gene expression levels to illustrate the predominantly temporal, with muted frontal and medial parietal, locations of the relative over-expression of genes associated with the diabetes and sex effect networks. The genes are selected to highlight insulin-dependent glucose transport (top left) and synaptic activity (bottom left) that occur in spatially overlapping patterns. By contrast, genes encoding for GLUT1 and GLUT3, present throughout virtually all neurons, show no meaningful regional variability in expression.

Spatially smoothed gene maps provided in previous work^48^ were used to visualize the spatial distributions of the genes in the targeted analysis and discovered in the unsupervised analysis contrasted with the spatial distribution of the control genes. The targeted pathway shows the insulin-sensitive glucose transporter pathway genes (*SLC2A4*, *SLC2A4RG*); the unsupervised pathway shows the distribution of GABAergic synapse related genes (*GABRA5*, *GABRB1*, *GLRA2*, *SLC12A2*); and the control glucose transporters show insulin-independent glucose transporters that are widely expressed in neurons and astrocytes (*SLC2A1*, *SLC2A3*). Visualizations involved normalizing the original maps to the unit range and averaging across genes, then plotting in on an average 3D surface using the *nilearn* package.^35^ All results of these analyses are illustrated in Fig. 5.

## Results

### Cohorts

The current study consisted of two imaging cohorts: a large cohort examined with 3T MRI and a complementary cohort scanned at ultrahigh-field 7T MRI. The 3T cohort consisted of a study of 1521 adults (829 males) aged 31–90 years old who received metabolic panels, cognitive assessment, *APOE* genotyping and extensive diabetes monitoring. The cohort was enriched for individuals carrying the APOE4 gene (437 APOE4+) and who had well-controlled diabetes (250 diabetic, 5.6% mean HbA1c). The cohort was balanced across sexes for APOE4+ status and age. Males were slightly more likely to be diabetic (19% versus 13%) and cognitively impaired (12% versus 8%) than females, although they had equivalent HbA1c values. To ensure that the sex effects noted in this dataset were not driven by small baseline variations in diabetes prevalence across sexes, we collected a second dataset of 100 individuals (52 males) scanned on 7T fMRI. To disambiguate the effects of sex from diabetes, this cohort was recruited to be healthy at baseline (diagnosis of diabetes was exclusionary) and cognitively unimpaired. Additionally, all individuals in the 7T cohort were scanned on two different days, allowing for the reproducibility of metrics to be assessed within individuals. Complete descriptive statistics of cohort characteristics can be found in Table 1. Both cohorts received resting-state fMRI scans, as detailed in the ‘Materials and Methods’ section.

### Age decreases functional network segregation

Functional network segregation was significantly decreased with age in both the 3T and 7T cohorts (Fig. 2). Network segregation was computed in 13 functionally defined networks^37^ in both resting-state fMRI datasets. All network ROIs are illustrated in Supplementary Fig. 1. Increased age robustly correlated with decreased functional connectivity in both the 3T (Fig. 2A) and 7T (Fig. 2B) datasets. Networks of interest for this paper (cingulo-opercular, default mode, salience and lateral somatomotor) were selected in a data-driven manner, as they were the only networks that robustly exhibited the main results in both 3T and 7T cohorts. All analyses are repeated for all networks, however, and are presented in the Supplementary figures. In the networks of interest for this paper, there are notable decreases in both datasets as age increases (*r* = [−0.26, −0.41] at 3T, *r* = [−0.27, 0.40] at 7T, *P* < 1e−6 throughout). We report the range of correlations as the [minimum, maximum] values across networks, with the full set of values in Fig. 2 (in this case) or Supplementary Table 1 as noted throughout the results.

In the 3T dataset, we observed this association of decreased network segregation with increased age throughout the brain (Supplementary Fig. 2), with most networks showing a prominent negative correlation (*r* = [−0.17, −0.41], *P* < 1e−16 throughout). Notable exceptions to this trend were reward (*r* = 0.03, *P* = 0.3) and dorsal somatomotor (*r* = 0.04, *P* = 0.4) networks; both have additional notes on preprocessing in Supplementary Methods. We also observe the association of decreased network segregation with increased age throughout the brain in the 7T dataset (Supplementary Fig. 3). All networks showed a significant negative correlation with age (*r* = [−0.23, −0.48], *P* < 0.01 throughout), although parieto-medial, medial temporal and dorsal somatomotor networks showed similar non-significant results as noted in the 3T dataset and Supplementary Methods. Acquiring two scans on different days in each individual in this dataset allowed us to further test the reproducibility of network segregation and the robustness of age-related effects. Network segregation was highly reproducible within individuals across scanning days (Supplementary Fig. 4; *r* = [0.58, 0.69], *P* < 0.001 for all networks of interest). Additionally, correcting age-related trends with random effect of individual repeated scans did not materially change the original results (Supplementary Table 1). Taken together, the 3T and 7T datasets show a robust and consistent negative association of age with network segregation throughout the brain.

### Males and participants with diabetes show decreased functional network segregation in overlapping spatial distributions

We next examined the relationship between diabetes status and network segregation across the lifespan (Fig. 3A). The 3T dataset included 250 individuals with diabetes, typically well-managed (see Table 1 and Supplementary Methods for clinical characterization). Consistent with results shown in the previous section, age generally correlated with a decrease in network segregation in participants with and without diabetes. However, participants with diabetes showed significantly lower network segregation than healthy participants in only a subset of networks, namely the cingulo-opercular, default mode, fronto-parietal, salience and lateral somatomotor networks (*t* = [−2.47, 5.18], *P*≤0.01 throughout). These networks showed a spatial susceptibility to loss of network segregation in diabetes beyond ageing; other networks did not show similar functional decreases in diabetes (Supplementary Fig. 5). Additionally, these effects were not a simple correlation of diabetes status with age, as they persisted in OLS models fit to account for age and diabetes simultaneously (Supplementary Table 2).

Similar to diabetes, males showed a greater decrease in network segregation in a spatially dependent manner compared to females. In the 3T dataset, male sex correlated with significantly decreased network segregation across the adult lifespan in the cingulo-opercular (*t* = −6.27, *P* < 0.001), default mode (*t* = −5.58, *P* < 0.001), salience (*t* = −3.50, *P* < 0.001) and lateral somatomotor (*t* = −2.76, *P* < 0.001) networks (Fig. 3B), as well as marginally increased network segregation in the auditory network (*t* = 1.98, *P* = 0.05). All other networks exhibited no sex effects (Supplementary Fig. 6). As with the diabetes analysis, we fit OLS models with sex and age simultaneously to ensure that sex effects were not confounded by age (Supplementary Table 3). To ensure that these effects were not driven by the prevalence of diabetes across sexes, we fit an OLS with main effects of both diabetes and sex and an interaction effect between them for each network (Supplementary Table 4). We observed independent main effects of sex and diabetes with no significant interaction effects, giving confidence that these effects are independent but spatially overlapping effects.

To further ensure these sex effects were independent of diabetes, we repeated the same analysis in the 7T cohort, which screened individuals to be free of diabetes at baseline. Each individual in this cohort received two resting-state fMRI scans on different days to further ensure the reproducibility of these results. Four networks showed the same spatial distribution of male sex correlated with decreased network segregation (Fig. 3C): cingulo-opercular (*t* = −2.37, *P* = 0.02), default mode (*t* = −3.71, *P* < 0.001), salience (*t* = −1.67, *P* = 0.1) and somatomotor lateral (*t* = 3.36, *P* < 0.001). Other networks showed no meaningful associations with sex effects (Supplementary Fig. 7). As in all other analyses, we analyzed these effects with an OLS to correct for age to ensure their independence of age (Supplementary Table 3).

To ensure that these sex differences are also not caused by differences in glucose dynamics below the threshold for diagnosis of diabetes, we ran two additional OLS models for each network in both datasets, where the regressor of interest was either fasting blood glucose (collected for 689 individuals in the 3T dataset and 99 individuals in the 7T dataset) or HbA1c (collected for 689 individuals in the 3T dataset and all individuals in the 7T dataset; see Supplementary Methods for clinical data notes) instead of diabetes status. These models included the biomarker (fasting blood glucose or HbA1c), sex and the interaction effect between the two to again test for any dependencies, as well as age (Supplementary Table 5). We observed interaction effects between fasting blood glucose and HbA1c with male sex only in the cingulo-opercular network and only in the 3T dataset, so there is no reproducible interaction between these biomarkers and male sex that drives the overlap between diabetes and male sex effects on network segregation.

Thus, there are four functional networks that exhibited *spatially coherent but independent* effects of diabetes and male sex (Fig. 3). These are the cingulo-opercular, default mode, salience and lateral somatomotor networks. Each of these networks exhibited greater decreases in functional segregation beyond normal ageing in individuals with diabetes, as well as exhibiting similar decreases in males independent of diabetes status. Taken together, these results indicate a potential predisposition to functional effects from endocrine pathways in these particular networks—an association that is further explored in light of APOE status and spatial transcriptomics in the next sections. Before moving on, it is interesting to note that, unlike these four networks, the fronto-parietal network shows a dichotomy between diabetes and sex. Decreased network segregation independent of age correlated with diabetes in the fronto-parietal network (Supplementary Fig. 5, panel 5), but not with sex in either the 3T (Supplementary Fig. 6, panel 5) or 7T (Supplementary Fig. 7, panel 5) datasets.

### APOE4+ individuals showed decreased functional network segregation in different regions than diabetes and male sex effects

Given previous work establishing that APOE4 is associated with a greater risk of Alzheimer’s disease in females,^49,50^ but potentially greater tau deposition and larger protective effects of APOE2 in males,^51,52^ we compared the effects of APOE4 positivity (single or double APOE ε4 allele) on network segregation effects in a similar manner to the diabetes and sex effects. We observed no broad association of APOE4 status with decreased network segregation throughout the brain (Supplementary Fig. 8), even when corrected for age as in the other analyses (Supplementary Table 6). Two networks exhibited greater functional decline in APOE4+ individuals (Fig. 4). We observed a modest decline in parieto-medial network segregation (*r* = −2.15, *P* = 0.03) and a marginal decline in the auditory network (*r* = 01.94, *P* = 0.05). It is interesting to note that the networks affected in APOE4+ individuals are spatially distinct from those identified as effected by diabetes and sex. While they undoubtedly lead to convergent effects in later-stage cognitive decline, the effects are spatially non-overlapping at the relatively early markers of functional decline studied in these datasets.

### Targeted and unsupervised spatial transcriptomic analyses identify pathways associated with diabetes and male sex network segregation effects

We used spatial transcriptomics to extend these results and explore what genetic pathways are expressed more in the networks that exhibited functional decline in these conditions. We conducted these analyses on transcriptomic data from six post-mortem brains provided by the Allen Human Brain Atlas.^31^ The measure of interest was the relative expression ratio of networks of interest (cingulo-opercular, default mode, salience and lateral somatomotor) compared to the rest of the functionally defined ROIs. We used this ratio to determine which genes were most significantly associated with the functional decline in diabetes and male sex. A relative expression level of 1 indicated the same level of expression (no association with networks of interest), while an expression significantly greater or less than 1 indicated relative over- or under-expression, respectively, of a gene in these networks compared to the rest of the brain. These analyses followed two analysis streams: a targeted set of genes selected on *a priori* hypotheses and an unsupervised approach based on analyzing relative expression levels of all available genes (14 609 genes in total).

In the targeted analysis, we tested three initial hypotheses: the over-expression of insulin-sensitive glucose transporters in regions affected by diabetes, the over-expression of estrogen receptors in regions adversely affected by male sex and the over-expression of metabotropic glutamate receptors (mGluR) in regions adversely affected by male sex. We hypothesized genes encoding insulin-sensitive glucose transporters (*SLC2A4*, *SLC2A4RG*, *SLC2A12*) would be over-expressed in the regions that experienced functional decline in diabetes, while other glucose transporters (e.g. *SLC2A1* and *SLC2A3*) that are broadly expressed in neurons would have an unrelated spatial distribution, as prior work has found that insulin-dependent glucose transport, particularly via GLUT4, supports functional neurometabolic demands.^53,54^ This hypothesis was borne out by the spatial transcriptomic data (Fig. 5A, upper panels, and Fig. 5B), with insulin-sensitive glucose transporters over-expressed (ratio = 1.37, [1.27, 1.51]) compared to other glucose transporters (ratio = 1.11, [0.92, 1.28]) in these networks. As estrogen presence, either through normal hormonal variation or hormone replacement therapy, has been associated with improved cognitive outcomes in ageing,^24-26^ we hypothesized that the genes encoding estrogen receptors (*ER1*, *GPER1*) would exhibit increased expression in the regions that showed a female protective effect when compared to selected control hormone receptors (inhibin receptors; *INHA*, *INHBA*, *INHBB*, *INHBC*) known to be widely expressed throughout the brain.^55^ While we did not find the control genes to be meaningfully associated with the selected networks (ratio = 1.02, [0.85, 1.29]), we also found the estrogen receptors to not be significantly increased in the networks of interest (ratio = 1.02, [1.01, 1.04]). We tested a final targeted hypothesis on mGluR over-expression in the regions exhibiting a potential female protective effect, as estrogen is known to modulate the activity of mGluRs.^56-58^ Broadly, we observed genes encoding for mGluRs (*GRM1–GRM8*) to be over-expressed in the regions exhibiting a female protective effect (ratio = 1.16, [1.01, 1.40]), especially when contrasted with serotonergic receptors that are expressed throughout the brain (ratio = 1.05, [0.85, 1.29]). As a summary of the targeted analysis results: while insulin-dependent glucose transport and mGluR expression levels were relatively over-expressed in the regions shown to be adversely affected by diabetes and male sex, the relative expression levels of estrogen receptors did not have a reliable association with these regions, indicating a functional role of hormones other than simple presence or absence.

To complement these targeted analyses, we performed an unsupervised analysis across the entire set of genes available through the Allen Human Brain Atlas to identify the pathways most significantly associated with the regions identified as susceptible to functional decline in diabetes and males. The expression ratio and relative significance level (with *P*-values adjusted via FDR correction) were computed independently for every gene available (Fig. 5B shows the entire volcano plot of genes with the targeted selection genes highlighted, while Fig. 5C shows a zoomed portion with genes highlighted by the unsupervised analysis as most significant). To aid in interpreting the genes most significantly associated with the networks affected by diabetes and male sex, we performed two additional analyses on the top 500 genes (selected by significance level): GSEA^45^ and pathway search via NDEx.^47^ NDEx results highlighted genes in insulin-signaling pathways in these regions (*ARHGAP33*, *MAPK7*, *PRKCD*, *TRIB3*, *TSC1*) and general pathways associated with Alzheimer’s disease (*PLCB2*, *SNCA*, *FZD1*). Interestingly, one gene was common between the insulin-signaling and Alzheimer’s disease-related pathways (*KIF5B*) and was relatively under-expressed in these networks. NDEx also highlighted synaptic signaling pathways associated with these networks, with GABAergic (both relative over- and under-expression of different genes) and dopaminergic (relative over-expression only) synapses in particular, and no serotonergic receptor genes noted (signifying they are valid control genes). Finally, the NDEx pathways showed a number of genes in the VEGFA–VEGFR2 pathway as associated with the spatial distribution of diabetes and sex effects noted above, with seven genes significantly over-expressed and two under-expressed in these regions. The spread of expression ratios of the genes highlighted by the NDEx analyses is shown in the bottom of Fig. 5A, with relative expression ratios and FDR-corrected significance levels shown in the zoomed volcano plot in Fig. 5C. We provide a complete list of all genes highlighted by these analyses with the pathways they belong to in Supplementary Table 7. GSEA revealed a similar pattern of related gene families (Fig. 5D), implicating a predominance of distal association (presynapse, synapse, somatodentritic compartment, neuron projection gene sets) as well as intercellular communication (cell–cell signaling gene set) in the pathways most associated with over-expression in these regions. The entire list of gene families as found by GSEA is provided in Supplementary Table 8.

The spatial distributions of expression of the insulin-dependent genes and GABAergic synapses illustrate how these processes overlap spatially independently of cell density (Fig. 5E). The spatially localized distributions of both diabetes-associated genes (*SLC2A4*, *SLC2A4RG*) and genes associated with GABAergic synapses (*GABRA5*, *GABRB1*, *GLRA2*) contrasted with control genes for glucose transporters (*GLUT1*, *GLUT3*) that are expressed broadly throughout the brain. Both the targeted and unsupervised gene selections showed a temporal predominance in expression, with medial frontal and parietal highlights as well. In contrast, the control visualization of insulin-independent glucose transporters showed a relatively uniform distribution throughout the brain. As a final control analysis for both the targeted and unsupervised analyses, we computed the relative expression levels of genes that are considered markers of cell density throughout the brain to demonstrate that the effects seen here are not driven by cell density (Fig. 5A, bottom panel; ratio = 0.95, [0.91, 1.24]). Discussion of the rationale for the selection of these control genes is provided in Supplementary Methods, and all expression ratios with associated *P*-values (both corrected and uncorrected) for the entire set of genes analyzed can be found in Supplementary Table 9.

In summary, the regions that exhibit spatially overlapping effects of diabetes and male sex (Fig. 3) showed a relative over-expression of genes associated with both insulin signaling and inter-neuron communication, especially dopaminergic and GABAergic signaling (Fig. 5). This is in contrast to no noticeable differential expression of estrogen receptors, indicating that although these regions exhibited a protective effect in females, this was not driven by baseline estrogen receptor density. The involvement of cell–cell signaling pathways, both endocrine and neuronal, further supported the hypothesis that these findings were driven by functional changes early in pathologic processes rather than end-stage structural degradation causing broad disruption. This was further supported by the finding that these regions also have baseline differences in the VEGF–VEGF2A expression levels, as vascular changes are also typically observed before structural integrity is compromised.

## Discussion

Together, our analyses suggest overlapping neuroendocrine and neurometabolic effects on functional decline during ageing. First, we showed a strong replication demonstrating increased age correlated with loss of network segregation, measured at both 3T and 7T resting-state fMRI, across a combined sample of more than 1600 individuals. This is an unsurprising finding given the prior evidence for this association,^15,30,59-62^ but nonetheless helpful to determine the utility of network segregation in this context, as it is a broad measure of functional dependence between regions.^63^ This also complements recent findings that functional connections that are metabolically expensive,^11,64^ especially over a longer distance,^14,63^ are weakened with age—a relationship that is captured by network segregation.^15^ As functional changes are some of the first to appear in age-related and neurodegenerative processes like Alzheimer’s disease,^8,9^ it is necessary to establish functional imaging biomarkers related to early pathological changes, both for basic research and for potential diagnostic purposes.

We also presented evidence of co-susceptibility to both diabetes and male sex effects on worsening network segregation beyond normal ageing in the cingulo-opercular, default mode, salience and lateral somatomotor networks. This co-localization unifies many previous threads of evidence into a cohesive picture of neuroendocrine coordination to maintain homeostasis. For example, there is prior evidence for task-specific sex differences in cingulo-opercular^61,65^ and salience^61,66^ network function, albeit often in the context of other neurological conditions.^66-68^ These effects are noted, however, without mention of metabolic dysregulation. Similarly, both type 1 diabetes^69^ and type 2 diabetes mellitus^10^ accelerate brain ageing, particularly in subcortical regions,^10^ but with limited assessment of region-specific functional deficits, particularly not assessing the combined effects of sex and diabetes in co-localized deficits. In this context, the evidence presented here is particularly striking, as it implicates the same regions as vulnerable to diabetes and male-specific insult or female-specific protective effects, discussed further below. We noted that the fronto-parietal network exhibited decreased functional segregation in diabetes but not in males, suggesting there may be effects unique to diabetes present in this network. There is some evidence for frontal-specific effects of type 2 diabetes mellitus,^10,70^ particularly in acute metabolic insult,^71^ but further research could elucidate why this network remains independent of male-specific functional decline as well.

One prominent potential mechanistic driver of sex-specific functional effects is the presence of the APOE4 mutation, known to drive sex-related differences in neuropathology.^72,73^ In the analyses presented here, however, the deleterious effects of APOE4 on network segregation were spatially distinct from the regions implicated in both sex and diabetes analyses and are confined to predominantly parieto-medial with some auditory network involvement. Given the relatively weak associations of APOE4 with functional decline overall, the evidence presented here does not offer much insight into the impact of APOE4 on the interplay between sex and diabetes effects and merely reaffirms its previously reported regional effects in the temporal and parietal lobes.^74,75^ These results do, however, remove APOE4 from consideration as a strong mechanistic driver of the effects observed in this study.

The spatial transcriptomic results we present, while not causative, provide several target markers for future exploration of mechanistic pathways underlying neuroendocrine feedback, particularly in the context of neurometabolic dysregulation. The relative over-expression of insulin-dependent glucose transporters in regions affected by diabetes reaffirms recent *in vitro* findings that implicate these transporters, especially GLUT4, in crucial supporting roles during periods of increased neurometabolic demand.^53,54^ The implication of insulin-signaling pathways in diabetes-related networks is perhaps unsurprising, but the genes that arose during the unsupervised analysis as having spatial co-localization with the networks associated with diabetes effects provide some interesting insights. The genes *PRKCD* and *TRIB3* in particular have been implicated in neurovascular complications in diabetes,^76,77^ but this specific spatial distribution has not previously been reported or analyzed. Similarly, lowered KIF5B has been associated *in vitro* with lowered dendritic function,^78^ synaptic plasticity,^79^ and membrane localization of GLUT4.^80^ The finding of lowered baseline expression of *KIF5B* in the regions affected by diabetes underscores its importance as a cofactor in insulin signaling, with its relative under-expression observed in regions exhibiting diabetes-specific functional deficits. These unsupervised analyses also provide increased evidence for the Wnt signaling pathway (specifically the finding of increased expression of *FZD1*), already frequently implicated in ageing and Alzheimer’s disease.^81^

Although the estrogen-protective effect has been discussed in previous literature,^24^ we did not observe an over-expression of estrogen receptors in the networks that exhibit greater functional decline in males. We did, however, observe relatively higher expression of mGluRs, which are known to have functional dependence on the presence of estrogen,^57,58^ indicating that the differences between male and female participants may be driven by functional pathways related to sex hormones without being directly tied to the hormone receptors. This was further supported by the implication of other specific neurotransmitter signaling pathways, particularly *GABRA5* over-expression, which has been associated with sexual dimorphisms in both GABAergic signaling and glucose regulation in mouse studies,^82^ even though it is equally expressed across sexes.^83^ Also associated with these regions were genes encoding dopaminergic synapse pathways, including *GRIA1* which is hypothesized to be functionally dependent on androgen hormones.^84^ These effects were not driven by simple increased neuronal or synaptic density, as both markers of neuronal density and serotonergic receptors did not differ between these networks and the rest of the brain (Fig. 5A). Finally, we noted that there are several genes from the VEGFA–VEGFR2 pathway over-expressed in these networks of interest at baseline. Although the mechanism of this angiogenesis pathway in supporting healthy brain ageing is incompletely characterized, emerging evidence has shown a striking sexual dimorphism in its expression during brain ageing, with females having a protective effect of increased angiogenesis up to age 75.^28^

In spite of the large sample sizes included in this study, there remain several limitations that should be addressed in future work, particularly if focused on personalized medicine. Network segregation, while a useful metric to assess age-related functional decline, was originally designed as a resting-state metric and is therefore limited in probing task-specific networks. It is also important to note that the datasets included here are cross-sectional by design and therefore are not as indicative of individual changes (although they are demonstrated here to be reproducible within individuals and demonstrated previously in Han *et al.*^85^). An ideal study to address both of these limitations would be a targeted set of task-based fMRI sequences to probe different circuits known to be impacted by ageing and repeated in the same individuals over a longer time period to assess how these metrics can change within individuals. Finally, it should be noted that while spatial transcriptomics provide an excellent sampling of baseline distributions of mRNA expression, protein expression levels fluctuate with age in specific patterns,^86,87^ and the inherently small sample size of the Allen Human Brain Atlas post-mortem cohort leaves the potential generalizability to larger populations uncertain.^31^ A larger and more thorough characterization of how these correlate with functional decline as shown here would look at fluid-based biomarkers to assess dynamic protein levels to complement the spatial transcriptomic maps.

The analyses we present here identified four key networks (cingulo-opercular, default mode, salience and lateral somatomotor) that showed particular vulnerability to both diabetes and male sex, independent of normal ageing effects. These findings were supported by spatial transcriptomic analyses showing over-expression of insulin-sensitive glucose transporters and dopaminergic and GABAergic receptors in these regions, while also highlighting the involvement of specific synaptic and vascular signaling pathways. Notably, while APOE4 status showed some influence on brain function, its effects were spatially distinct from the diabetes and sex-specific patterns observed. The overlap between diabetes and male sex effects in specific brain networks, along with the identification of relevant genetic pathways, provides new insights into the co-localized neuroendocrine and neurometabolic mechanisms of functional decline in ageing and suggest potential targets for personalized interventions based on metabolic and endocrine profiles.

## Supplementary Material

fcag347_Supplementary_Data

## Data Availability

The 3T fMRI dataset used in these analyses is part of the Mayo Clinic Study of Aging, and the data are publicly accessible upon reasonable request to the Mayo Clinic Study of Aging. The 7T fMRI dataset used in these analyses is part of the Protecting the Aging Brain dataset, which is provided by the authors at openneuro.org/datasets/ds005405. All spatial transcriptomic data are provided with open access by the Allen Institute at https://human.brain-map.org. Processed spatial transcriptomic maps in Montreal Neurological Institute space and parcellated for FreeSurfer have also been provided at https://www.meduniwien.ac.at/neuroimaging/mRNA.html. All fMRI preprocessing and statistical results are done using open-source packages as described in the ‘Materials and Methods’ section, with their appropriate source code locations cited, and all results from custom analyses (e.g. relative gene expression ratios in particular networks) are included as Supplementary data files.

## References

[1] Bobba-Alves N, Juster R-P, Picard M. The energetic cost of allostasis and allostatic load. Psychoneuroendocrinology. 2022;146:105951. 10.1016/j.psyneuen.2022.105951 PMC10082134 36302295

[2] Bobba-Alves N, Sturm G, Lin J, et al Cellular allostatic load is linked to increased energy expenditure and accelerated biological aging. Psychoneuroendocrinology. 2023;155:106322. 10.1016/j.psyneuen.2023.106322 PMC10528419 37423094

[3] Kraft TS, Venkataraman VV, Wallace IJ, et al The energetics of uniquely human subsistence strategies. Science. 2021;374:eabf0130. 10.1126/science.abf0130 34941390

[4] McKhann GM, Knopman DS, Chertkow H, et al The diagnosis of dementia due to Alzheimer’s disease: Recommendations from the National Institute on Aging-Alzheimer’s ASSOCiation workgroups on diagnostic guidelines for Alzheimer’s disease. Alzheimers Dement. 2011;7:263–269. 10.1016/j.jalz.2011.03.005 PMC3312024 21514250

[5] O’Brien JT, Thomas A. Vascular dementia. The Lancet. 2015;386:1698–1706. 10.1016/S0140-6736(15)00463-8 26595643

[6] Villain N, Fouquet M, Baron J-C, et al Sequential relationships between grey matter and white matter atrophy and brain metabolic abnormalities in early Alzheimer’s disease. Brain. 2010;133:3301–3314. 10.1093/brain/awq203 PMC3291528 20688814

[7] Brickman AM, Provenzano FA, Muraskin J, et al Regional white matter hyperintensity volume, not hippocampal atrophy, predicts incident Alzheimer disease in the community. Arch Neurol. 2012;69:1621–1627. 10.1001/archneurol.2012.1527 PMC3597387 22945686

[8] Lee J, Burkett BJ, Min H-K, et al Synthesizing images of tau pathology from cross-modal neuroimaging using deep learning. Brain. 2024;147:980–995. 10.1093/brain/awad346 PMC10907092 37804318

[9] Jack CR, Knopman DS, Jagust WJ, et al Update on hypothetical model of Alzheimer’s disease biomarkers. Lancet Neurol. 2013;12:207–216. 10.1016/S1474-4422(12)70291-0 PMC3622225 23332364

[10] Antal B, McMahon LP, Sultan SF, eds, et al Type 2 diabetes mellitus accelerates brain aging and cognitive decline: Complementary findings from UK Biobank and meta-analyses. eLife. 2022;11:e73138. 10.7554/eLife.73138 PMC9132576 35608247

[11] Weistuch C, Mujica-Parodi LR, Razban RM, et al Metabolism modulates network synchrony in the aging brain. Proc Natl Acad Sci U S A. 2021;118:e2025727118. 10.1073/pnas.2025727118 PMC8501850 34588302

[12] Mujica-Parodi LR, Amgalan A, Sultan SF, et al Diet modulates brain network stability, a biomarker for brain aging, in young adults. Proc Natl Acad Sci U S A. 2020;117:6170–6177. 10.1073/pnas.1913042117 PMC7084077 32127481

[13] Haas SS, Abbasi F, Watson K, et al Metabolic status modulates global and local brain age estimates in overweight and obese adults. Biol Psychiatry Cogn Neurosci Neuroimaging. 2025;10:278–285. 10.1016/j.bpsc.2024.11.017 PMC11890935 39615789

[14] Tomasi D, Volkow ND. Aging and functional brain networks. Mol Psychiatry. 2012;17:471–558. 10.1038/mp.2011.81 PMC3193908 21727896

[15] Manza P, Wiers CE, Shokri-Kojori E, et al Brain network segregation and glucose energy utilization: Relevance for age-related differences in cognitive function. Cereb Cortex. 2020;30:5930–5942. 10.1093/cercor/bhaa167 PMC7673478 32564073

[16] Antal BB, van Nieuwenhuizen H, Chesebro AG, et al Brain aging shows nonlinear transitions, suggesting a midlife ‘critical window’ for metabolic intervention. Proc Natl Acad Sci U S A. 2025;122:e2416433122. 10.1073/pnas.2416433122 PMC11912423 40030017

[17] Scarmeas N, Luchsinger JA, Schupf N, et al Physical activity, diet, and risk of Alzheimer disease. JAMA. 2009;302:627–637. 10.1001/jama.2009.1144 PMC2765045 19671904

[18] de la Monte SM, Wands JR. Alzheimer’s disease is type 3 diabetes – Evidence reviewed. J Diabetes Sci Technol. 2008;2:1101–1113. 10.1177/193229680800200619 PMC2769828 19885299

[19] Chatterjee S, Peters SAE, Woodward M, et al Type 2 diabetes as a risk factor for dementia in women compared with men: A pooled analysis of 2.3 million people comprising more than 100,000 cases of dementia. Diabetes Care. 2016;39:300–307. 10.2337/dc15-1588 PMC4722942 26681727

[20] Ho N, Sommers MS, Lucki I. Effects of diabetes on hippocampal neurogenesis: Links to cognition and depression. Neurosci Biobehav Rev. 2013;37:1346–1362. 10.1016/j.neubiorev.2013.03.010 PMC3788092 23680701

[21] Kaiser AB, Zhang N, Der Pluijm WV. Global prevalence of type 2 diabetes over the next ten years (2018–2028). Diabetes. 2018;67:202-LB.

[22] Levine DA, Gross AL, Briceño EM, et al Sex differences in cognitive decline among US adults. JAMA Netw Open. 2021;4:e210169. 10.1001/jamanetworkopen.2021.0169 PMC7907956 33630089

[23] Barth C, Crestol A, de Lange A-MG, Galea LAM. Sex steroids and the female brain across the lifespan: Insights into risk of depression and Alzheimer’s disease. Lancet Diabetes Endocrinol. 2023;11:926–941. 10.1016/S2213-8587(23)00224-3 37865102

[24] Russell JK, Jones CK, Newhouse PA. The role of estrogen in brain and cognitive aging. Neurotherapeutics. 2019;16:649–665. 10.1007/s13311-019-00766-9 PMC6694379 31364065

[25] Genazzani AR, Pluchino N, Luisi S, Luisi M. Estrogen, cognition and female ageing. Hum Reprod Update. 2007;13:175–187. 10.1093/humupd/dml042 17135285

[26] Saleh RNM, Hornberger M, Ritchie CW, Minihane AM. Hormone replacement therapy is associated with improved cognition and larger brain volumes in at-risk APOE4 women: Results from the European Prevention of Alzheimer’s Disease (EPAD) cohort. Alzheimers Res Ther. 2023;15:10. 10.1186/s13195-022-01121-5 PMC9830747 36624497

[27] Oveisgharan S, Arvanitakis Z, Yu L, Farfel J, Schneider JA, Bennett DA. Sex differences in Alzheimer’s disease and common neuropathologies of aging. Acta Neuropathol. 2018;136:887–900. 10.1007/s00401-018-1920-1 PMC6279593 30334074

[28] Torres-Espin A, Radabaugh HL, Treiman S, et al Sexually dimorphic differences in angiogenesis markers are associated with brain aging trajectories in humans. Sci Transl Med. 2024;16:eadk3118. 10.1126/scitranslmed.adk3118 PMC12092094 39602511

[29] Lopez-Lee C, Torres ERS, Carling G, Gan L. Mechanisms of sex differences in Alzheimer’s disease. Neuron. 2024;112:1208–1221. 10.1016/j.neuron.2024.01.024 PMC11076015 38402606

[30] Chan MY, Park DC, Savalia NK, Petersen SE, Wig GS. Decreased segregation of brain systems across the healthy adult lifespan. Proc Natl Acad Sci U S A. 2014;111:E4997–E5006. 10.1073/pnas.1415122111 PMC4246293 25368199

[31] Hawrylycz MJ, Lein ES, Guillozet-Bongaarts AL, et al An anatomically comprehensive atlas of the adult human brain transcriptome. Nature. 2012;489:391–399. 10.1038/nature11405 PMC4243026 22996553

[32] Roberts RO, Geda YE, Knopman DS, et al The Mayo Clinic study of aging: Design and sampling, participation, baseline measures and sample characteristics. Neuroepidemiology. 2008;30:58–69. 10.1159/000115751 PMC2821441 18259084

[33] Kumar R, Tan L, Kriegstein A, et al Ground-truth ‘resting-state’ signal provides data-driven estimation and correction for scanner distortion of fMRI time-series dynamics. Neuroimage. 2021;227:117584. 10.1016/j.neuroimage.2020.117584 PMC12997525 33285328

[34] Esteban O, Markiewicz CJ, Blair RW, et al fMRIPrep: A robust preprocessing pipeline for functional MRI. Nat Methods. 2019;16:111–116. 10.1038/s41592-018-0235-4 PMC6319393 30532080

[35] Abraham A, Pedregosa F, Eickenberg M, et al Machine learning for neuroimaging with scikit-learn. Front Neuroinform. 2014;8:14. 10.3389/fninf.2014.00014 PMC3930868 24600388

[36] Wang S, Peterson DJ, Gatenby JC, Li W, Grabowski TJ, Madhyastha TM. Evaluation of field map and nonlinear registration methods for correction of susceptibility artifacts in diffusion MRI. Front Neuroinform. 2017;11:17. 10.3389/fninf.2017.00017 PMC5318394 28270762

[37] Seitzman BA, Gratton C, Marek S, et al A set of functionally-defined brain regions with improved representation of the subcortex and cerebellum. NeuroImage. 2020;206:116290. 10.1016/j.neuroimage.2019.116290 PMC6981071 31634545

[38] Power JD, Plitt M, Gotts SJ, et al Ridding fMRI data of motion-related influences: Removal of signals with distinct spatial and physical bases in multiecho data. Proc Natl Acad Sci U S A. 2018;115:E2105–E2114. 10.1073/pnas.1720985115 PMC5834724 29440410

[39] Rubinov M, Sporns O. Weight-conserving characterization of complex functional brain networks. NeuroImage. 2011;56:2068–2079. 10.1016/j.neuroimage.2011.03.069 21459148

[40] Sidhu AS, Duarte KTN, Shahid TH, et al Age- and sex-specific patterns in adult brain network segregation. Hum Brain Mapp. 2025;46:e70169. 10.1002/hbm.70169 PMC11907239 40084534

[41] Hagenauer MH, Sannah Y, Hebda-Bauer EK, et al Resource: A curated database of brain-related functional gene sets (Brain.GMT). MethodsX. 2024;13:102788. 10.1016/j.mex.2024.102788 PMC11267058 39049932

[42] Markello RD, Hansen JY, Liu Z-Q, et al Neuromaps: Structural and functional interpretation of brain maps. Nat Methods. 2022;19:1472–1479. 10.1038/s41592-022-01625-w PMC9636018 36203018

[43] Burt JB, Helmer M, Shinn M, Anticevic A, Murray JD. Generative modeling of brain maps with spatial autocorrelation. NeuroImage. 2020;220:117038. 10.1016/j.neuroimage.2020.117038 32585343

[44] Benjamini Y, Hochberg Y. Controlling the false discovery rate: A practical and powerful approach to multiple testing. J Royal Stat Soc Ser B. 1995;57:289–300.

[45] Subramanian A, Tamayo P, Mootha VK, et al Gene set enrichment analysis: A knowledge-based approach for interpreting genome-wide expression profiles. Proc Natl Acad Sci U S A. 2005;102:15545–15550. 10.1073/pnas.0506580102 PMC1239896 16199517

[46] Liberzon A, Birger C, Thorvaldsdóttir H, Ghandi M, Mesirov JP, Tamayo P. The Molecular Signatures Database (MSigDB) hallmark gene set collection. Cell Syst. 2015;1:417–425. 10.1016/j.cels.2015.12.004 PMC4707969 26771021

[47] Pillich RT, Chen J, Churas C, et al NDEx: Accessing network models and streamlining network biology workflows. Curr Protoc. 2021;1:e258. 10.1002/cpz1.258 PMC8544027 34570431

[48] Gryglewski G, Seiger R, James GM, et al Spatial analysis and high resolution mapping of the human whole-brain transcriptome for integrative analysis in neuroimaging. Neuroimage. 2018;176:259–267. 10.1016/j.neuroimage.2018.04.068 29723639

[49] Altmann A, Tian L, Henderson VW, Greicius MD. Sex modifies the APOE-related risk of developing Alzheimer’s disease. Ann Neurol. 2014;75:563–573. 10.1002/ana.24135 PMC4117990 24623176

[50] Walters S, Contreras AG, Eissman JM, et al Associations of sex, race, and apolipoprotein E alleles with multiple domains of cognition among older adults. JAMA Neurol. 2023;80:929–939. 10.1001/jamaneurol.2023.2169 PMC10352930 37459083

[51] Wood ME, Xiong LY, Wong YY, et al Sex differences in associations between APOE ε2 and longitudinal cognitive decline. Alzheimers Dement. 2023;19:4651–4661. 10.1002/alz.13036 PMC10544702 36994910

[52] Yan S, Zheng C, Paranjpe MD, et al Sex modifies APOE ε4 dose effect on brain tau deposition in cognitively impaired individuals. Brain. 2021;144:3201–3211. 10.1093/brain/awab160 PMC8634082 33876815

[53] Ashrafi G, Wu Z, Farrell RJ, Ryan TA. GLUT4 mobilization supports energetic demands of active synapses. Neuron. 2017;93:606–615.e3. 10.1016/j.neuron.2016.12.020 PMC5330257 28111082

[54] Kula B, Antal B, Weistuch C, et al D-ꞵ-hydroxybutyrate stabilizes hippocampal CA3-CA1 circuit during acute insulin resistance. PNAS Nexus. 2024;3:pgae196. 10.1093/pnasnexus/pgae196 PMC11138115 38818236

[55] Namwanje M, Brown CW. Activins and inhibins: Roles in development, physiology, and disease. Cold Spring Harb Perspect Biol. 2016;8:a021881. 10.1101/cshperspect.a021881 PMC4930927 27328872

[56] Dewing P, Boulware MI, Sinchak K, Christensen A, Mermelstein PG, Micevych P. Membrane estrogen receptor-α interactions with metabotropic glutamate receptor 1a modulate female sexual receptivity in rats. J Neurosci. 2007;27:9294–9300. 10.1523/JNEUROSCI.0592-07.2007 PMC2904398 17728443

[57] Grove-Strawser D, Boulware MI, Mermelstein PG. Membrane estrogen receptors activate the metabotropic glutamate receptors mGluR5 and mGluR3 to bidirectionally regulate CREB phosphorylation in female rat striatal neurons. Neuroscience. 2010;170:1045–1055. 10.1016/j.neuroscience.2010.08.012 PMC2949475 20709161

[58] Fagan MP, Ameroso D, Meng A, Rock A, Maguire J, Rios M. Essential and sex-specific effects of mGluR5 in ventromedial hypothalamus regulating estrogen signaling and glucose balance. Proc Natl Acad Sci U S A. 2020;117:19566–19577. 10.1073/pnas.2011228117 PMC7430975 32719118

[59] Filippi M, Cividini C, Basaia S, et al Age-related vulnerability of the human brain connectome. Mol Psychiatry. 2023;28:5350–5358. 10.1038/s41380-023-02157-1 PMC11041755 37414925

[60] Argiris G, Stern Y, Habeck C. Cross-sectional and longitudinal age-related disintegration in functional connectivity: Reference ability neural network cohort. J Cogn Neurosci. 2024;36:2045–2066. 10.1162/jocn_a_02188 PMC11498141 38739573

[61] Ballard HK, Jackson TB, Symm AC, Hicks TH, Bernard JA. Age-related differences in functional network segregation in the context of sex and reproductive stage. Hum Brain Mapp. 2023;44:1949–1963. 10.1002/hbm.26184 PMC9980887 36541480

[62] Stanford W, Mucha PJ, Dayan E. Age-related differences in network controllability are mitigated by redundancy in large-scale brain networks. Commun Biol. 2024;7:701. 10.1038/s42003-024-06392-2 PMC11161655 38849512

[63] Razban RM, Antal BB, Dill KA, Mujica-Parodi LR. Brain signaling becomes less integrated and more segregated with age. Netw Neurosci. 2024;8:1051–1064. 10.1162/netn_a_00389 PMC11674493 39735489

[64] Deery HA, Liang EX, Siddiqui MN, et al Reconfiguration of metabolic connectivity in ageing. Commun Biol. 2024;7:1600. 10.1038/s42003-024-07223-0 PMC11608353 39616242

[65] Stoica T, Knight LK, Naaz F, Patton SC, Depue BE. Gender differences in functional connectivity during emotion regulation. Neuropsychologia. 2021;156:107829. 10.1016/j.neuropsychologia.2021.107829 33744320

[66] Zielinski BA, Andrews DS, Lee JK, et al Sex-dependent structure of socioemotional salience, executive control, and default mode networks in preschool-aged children with autism. NeuroImage. 2022;257:119252. 10.1016/j.neuroimage.2022.119252 PMC11107798 35500808

[67] Jones DT, Knopman DS, Gunter JL, et al Cascading network failure across the Alzheimer’s disease spectrum. Brain. 2016;139:547–562. 10.1093/brain/awv338 PMC4805086 26586695

[68] Jones DT, Machulda MM, Vemuri P, et al Age-related changes in the default mode network are more advanced in Alzheimer disease. Neurology. 2011;77:1524–1531. 10.1212/WNL.0b013e318233b33d PMC3198977 21975202

[69] Habes M, Jacobson AM, Braffett BH, et al Patterns of regional brain atrophy and brain aging in middle- and older-aged adults with type 1 diabetes. JAMA Netw Open. 2023;6:e2316182. 10.1001/jamanetworkopen.2023.16182 PMC10236234 37261829

[70] Choi SE, Roy B, Freeby M, Mullur R, Woo MA, Kumar R. Prefrontal cortex brain damage and glycemic control in patients with type 2 diabetes. J Diabetes. 2020;12:465–473. 10.1111/1753-0407.13019 PMC7210044 31886635

[71] Backeström A, Papadopoulos K, Eriksson S, et al Acute hyperglycaemia leads to altered frontal lobe brain activity and reduced working memory in type 2 diabetes. PLoS One. 2021;16:e0247753. 10.1371/journal.pone.0247753 PMC7978337 33739980

[72] Sundermann EE, Tran M, Maki PM, Bondi MW. Sex differences in the association between apolipoprotein E ε4 allele and Alzheimer’s disease markers. Alzheimers Dement (Amst). 2018;10:438–447. 10.1016/j.dadm.2018.06.004 PMC6120724 30182053

[73] Damoiseaux JS, Seeley WW, Zhou J, et al Gender modulates the APOE ε4 effect in healthy older adults: Convergent evidence from functional brain connectivity and spinal fluid tau levels. J Neurosci. 2012;32:8254–8262. 10.1523/JNEUROSCI.0305-12.2012 PMC3394933 22699906

[74] Turney IC, Chesebro AG, Rentería MA, et al APOE ε4 and resting-state functional connectivity in racially/ethnically diverse older adults. Alzheimers Dement (Amst). 2020;12:e12094. 10.1002/dad2.12094 PMC7508460 32999914

[75] Machulda MM, Jones DT, Vemuri P, et al Effect of APOE ε4 Status on intrinsic network connectivity in cognitively normal elderly subjects. Arch Neurol. 2011;68:1131–1136. 10.1001/archneurol.2011.108 PMC3392960 21555604

[76] Geraldes P, Hiraoka-Yamamoto J, Matsumoto M, et al Activation of PKCδ and SHP1 by hyperglycemia causes vascular cell apoptosis and diabetic retinopathy. Nat Med. 2009;15:1298–1306. 10.1038/nm.2052 PMC3290906 19881493

[77] Lu G, Li J, Gao T, et al Integration of dietary nutrition and TRIB3 action into diabetes mellitus. Nutr Rev. 2024;82:361–373. 10.1093/nutrit/nuad056 PMC10859691 37226405

[78] Fok AHK, Huang Y, So BWL, et al KIF5B plays important roles in dendritic spine plasticity and dendritic localization of PSD95 and FMRP in the mouse cortex *in vivo*. Cell Rep. 2024;43:113906. 10.1016/j.celrep.2024.113906 38451812

[79] Zhao J, Fok AHK, Fan R, eds, et al Specific depletion of the motor protein KIF5B leads to deficits in dendritic transport, synaptic plasticity and memory. eLife. 2020;9:e53456. 10.7554/eLife.53456 PMC7028368 31961321

[80] Semiz S, Park JG, Nicoloro SMC, et al Conventional kinesin KIF5B mediates insulin-stimulated GLUT4 movements on microtubules. EMBO J. 2003;22:2387–2399. 10.1093/emboj/cdg237 PMC155995 12743033

[81] Palomer E, Martín-Flores N, Jolly S, et al Epigenetic repression of Wnt receptors in AD: A role for Sirtuin2-induced H4K16ac deacetylation of Frizzled1 and Frizzled7 promoters. Mol Psychiatry. 2022;27:3024. 10.1038/s41380-022-01492-z PMC9205772 35296808

[82] Pei Z, He Y, Bean JC, et al Gabra5 plays a sexually dimorphic role in POMC neuron activity and glucose balance. Front Endocrinol (Lausanne). 2022;13:889122. 10.3389/fendo.2022.889122 PMC9471380 36120438

[83] Rau AR, Hentges ST. The relevance of AgRP neuron-derived GABA inputs to POMC neurons differs for spontaneous and evoked release. J Neurosci. 2017;37:7362–7372. 10.1523/JNEUROSCI.0647-17.2017 PMC5546108 28667175

[84] Trabzuni D, Ramasamy A, Imran S, et al Widespread sex differences in gene expression and splicing in the adult human brain. Nat Commun. 2013;4:2771. 10.1038/ncomms3771 PMC3868224 24264146

[85] Han L, Chan MY, Agres PF, Winter-Nelson E, Zhang Z, Wig GS. Measures of resting-state brain network segregation and integration vary in relation to data quantity: Implications for within and between subject comparisons of functional brain network organization. Cereb Cortex. 2024;34:bhad506. 10.1093/cercor/bhad506 PMC10883417 38385891

[86] Lehallier B, Gate D, Schaum N, et al Undulating changes in human plasma proteome profiles across the lifespan. Nat Med. 2019;25:1843–1850. 10.1038/s41591-019-0673-2 PMC7062043 31806903

[87] Shen X, Wang C, Zhou X, et al Nonlinear dynamics of multi-omics profiles during human aging. Nat Aging. 2024;4:1619–1634. 10.1038/s43587-024-00692-2 PMC11564093 39143318

