# Supplementary material for: Spatially convergent functional MRI signatures of diabetes and male sex identify genetic vulnerabilities to accelerated brain ageing
Source: Brain Commun. 2026 Sep 19;8(5):fcag347. doi: 10.1093/braincomms/fcag347 (PMC13599025; doi:10.1093/braincomms/fcag347)
Supplement: Supplementary file 1 [file fcag347_supplementary_data.zip › Supplementary_material_including_Supplementary_figures.docx]

**Supplementary materials for: Spatially convergent fMRI signatures of male sex and diabetes identify genetic vulnerabilities to accelerated brain aging**

**Supplemental methods**

*Clinical data collection*

For Table 1, not all participants in the 3T dataset received fasting blood glucose HbA1c measurements. The entire sample was 689 (372 males), with the statistics as noted in Table 1. All individuals in the 3T dataset had diabetes status confirmed by clinical diagnosis, with 244 individuals (156 male) confirmed to have Type 2 diabetes, and 6 individuals (4 male) confirmed to have Type 1 diabetes. For the 7T dataset, participants were recruited from the Boston metropolitan area through advertisements. Subject were excluded if they had contraindications to MRI, neurological or psychiatric conditions, a history of brain injury, insulin resistance, diabetes mellitus, recreational drug usage, excessive alcohol usage, and recent adherence to low-carbohydrate diets. All scans were conducted during morning hours, and participants were instructed to arrive after overnight fasting. All individuals were tested for HbA1c, but one participant did not complete the fasting blood glucose measurement as it was part of an oral glucose tolerance test, and they were averse to repeated blood draws over the two hour time course. In the 3T dataset, cognitive status was assessed clinically as cognitively normal, mild cognitive impairment, or dementia. In the cognitively impaired group presented in these analyses, 141 individuals (88 male) had mild cognitive impairment, and 16 individuals (13 male) had dementia. As so few individuals had dementia, the latter two groups were combined into a single group of cognitive impairment in these analyses. In the 7T dataset, all participants were confirmed to be cognitively normal using the CNS Vital Signs neurocognitive test battery *(1)*.

*Selection of control genes for cell density*

A potential concern in any spatial transcriptomics approach is that any spatial distribution of pathology is driven simply by cell density; that is, any process could be more correlated with regions where more cells are present. To ensure both targeted and unsupervised analyses presented in this work were not biased by relative cell density, five control genes were selected to provide a range of values for overall cellular density: three markers of neuron density (*RBFOX3, TH, MAP2*) and two markers of glial density (*GFAP, S100B*). *RBFOX3* was selected as a marker of neuron density, as it encodes for NeuN which is typically found in all neurons *(2)*. *TH* was selected as an additional marker of neuron density typically associated with interneurons in humans *(3)*. *MAP2* was selected as a tertiary marker of neuron density, with a particular emphasis on dendritic density *(5, 6)*. *GFAP* was selected as a general marker of (mature) astrocyte density *(6, 7)*. *S100B* was selected as a secondary marker of astrocyte density *(8)* and as a marker of oligodendrocyte density *(9)*. The relative expression levels of these proteins are shown in the bottom of Figure 4A to provide a comparison for the main results. They are also listed in the complete list of genes for that figure provided in Supplementary Table 7, and their exact expression ratios and associated significance levels are provided in Supplementary Table 9.

*Gene expression analyses*

Regional microarray expression data were obtained from six post-mortem brains (one female, 24–57 years old, 42.5±3.4) provided by the Allen Human Brain Atlas *(10)* (AHBA, <https://human.brain-map.org>). Data were processed with the *abagen* toolbox (version 0.1.3; <https://github.com/rmarkello/abagen>) using a 300-region functionally-defined volumetric atlas in MNI space *(11)*. Microarray probes were reannotated using data provided by *(12)*; probes not matched to a valid Entrez ID were discarded. Next, probes were filtered based on their expression intensity relative to background noise *(13)*, such that probes with intensity less than the background in >=20% of samples across donors were discarded. When multiple probes indexed the expression of the same gene, the probe with the most consistent pattern of regional variation across donors (i.e., differential stability; *(14)*) was selected, calculated with:

$$\Delta s\left( p \right)=\frac{1}{\left( \begin{matrix} N \\ 2 \end{matrix} \right)}\sum_{i=1}^{N-1} \sum_{j=i+1}^{N} \rho[B_{i}\left( p \right),B_{j}(p)]$$

where $\rho$ is Spearman’s rank correlation of the expression of a single probe $p$ across regions in two donor brains $B_{i}$ and $B_{j}$ and $N$ is the total number of donors. Regions correspond to the structural designations provided in the ontology from the AHBA.

The MNI coordinates of tissue samples were updated to those generated via non-linear registration using the Advanced Normalization Tools (ANTs *(15)*; <https://github.com/chrisfilo/alleninf>). Samples were assigned to brain regions in the provided atlas if their MNI coordinates were within 2mm of a given parcel. To reduce the potential for misassignment, sample-to-region matching was constrained by hemisphere and gross structural divisions (i.e., cortex, subcortex/brainstem, and cerebellum; *(12)*). All tissue samples not assigned to a brain region were discarded. Additionally, due to the inherent limitations of the AHBA in normalizing across subcortical and cortical samples *(16)*, ROIs that fell in deep or subcortical or cerebellar regions were omitted from further analyses (see Supplementary Figure 1 for all ROIs). This selection step was done by using only functional ROIs that overlapped with cortical structures in the Automated Anatomical Labeling atlas *(17)*.

Inter-subject variation was addressed by normalizing tissue sample expression values across genes using a robust sigmoid function *(18)*:

$$x_{\text{norm}}=\frac{1}{1+\exp\left( -\frac{x-\left\langle x \right\rangle}{\text{IQR}_{x}} \right)}$$

where $\left\langle x \right\rangle$ is the median and $\text{IQR}_{x}$ is the normalized interquartile range of the expression of a single tissue sample across genes. Normalized expression values were then rescaled to the unit interval:

$$x_{\text{scaled}}=\frac{x_{\text{norm}}-min(x_{\text{norm}})}{\max\left( x_{\text{norm}} \right)-min(x_{\text{norm}})}$$

Gene expression values were then normalized across tissue samples using an identical procedure. Samples assigned to the same brain region were averaged separately for each donor and then across donors.

*Gene set enrichment analyses*

For the unsupervised gene analyses, the top 500 most statistically significant genes (both over and under-expressed in the diabetes and sex-specific effect networks) were used in a gene set enrichment analysis (GSEA). Significance was determined by the FDR-corrected p-value derived using the null spatial maps as described in the main methods. This selection step was done as 500 is the maximum number of genes allowed in the online tool. Standard GSEA software ([https://www.gsea-msigdb.org](https://www.gsea-msigdb.org/); *(19)*) was used for this analysis, specifically focused on C5 (ontology gene sets) overlap from the molecular signatures database (MSigDB; *(20)*). Interested readers can replicate this result using the same online tool (<https://www.gsea-msigdb.org/gsea/msigdb/index.jsp>), which requires a brief account creation before access. The “Investigate” portion of the tool can be used to input the genes of interest (provided in Supplementary Table 9) and computing the overlaps with the C5 (ontology gene sets). The overlaps and significance values of the top 10 gene sets identified in this approach are provided in Supplementary Table 8, and visualized in Figure 4D. This unsupervised approach was extended using the same 500 genes as a query for related pathways using the Network Data Exchange (NDEx; *(21)*). Interested readers can use the same tool as linked above, but repeating the process using the “Query NDEx” option within “Investigate.” As the 500 gene cutoff is arbitrary, we have provided alternative versions of Figure 4D in Supplementary Figure 9b-c, but with cutoffs at 400 and 300 most significant genes instead to demonstrate that the results do not substantially change based on the cutoff.

*fMRI analyses*

There are a few additional notes regarding specific functional network to aid in interpreting the supplemental figures. Three networks in particular have noticeably fewer (<10 total) ROIs compared to the other functional networks; these are the reward, parieto-medial, and medial temporal networks. Because of this, they are subject to more individual variability in the segregation metric and exhibit noisier, and therefore weaker, associations. Also, the somatomotor dorsal ROIs frequently have overlap with non-brain areas particularly in older brains with volume loss, so the signals from these ROIs are often noisier and must be excluded in the processing pipeline used here, leading to far fewer usable participants in this network. Readers are therefore urged to interpret the results associated with these networks with care, and for this reason these networks largely go un-emphasized in the main text. In addition to these considerations, we also demonstrate the validity of the motion correction in the fMRI preprocessing pipeline employed in this paper by showing summary metrics in Supplementary Figures 10-11.

*Statistical analyses*

There is one cosmetic note for statistics in the supplemental sections: in Supplementary Tables 1-7, p = 0 means that p < 1e-4. This choice was made to reduce the size of the tables to be more legible as very small p-values are uninformative and are typically tied to the size of the dataset. All p-values greater than this cutoff are explicitly written in the tables.

Supplementary Figures

**Supplementary Figure 1**: Spatial visualizations for all networks within the atlas used for the analyses presented here (as referenced in Figure 2 and the main text). Certain ROIs present in the original atlas have been excluded from these analyses; these include ROIs not assigned to a specific network (12 ROIs total) and ROIs that lie in the cerebellum (26 ROIs total).

**Supplementary Figure 2**: **Loss of functional network segregation is associated with age throughout the brain when examined with 3T fMRI**. This figure extends Figure 2A to all the networks examined in these analyses. Individual points represent segregation computed from each fMRI scan. Lines represent linear regression between segregation and age with 68^th^ percentile bootstrapped confidence interval shaded.

**Supplementary Figure 3**: **Loss of functional network segregation is associated with age throughout the brain when examined with 7T fMRI**. This figure extends Figure 2B to all the networks examined in these analyses. Individual points represent segregation computed from each fMRI scan. Lines represent linear regression between segregation and age with 68^th^ percentile bootstrapped confidence interval shaded.

**Supplementary Figure 4**: **Network segregation is highly correlated within individuals across different scanning days.** Each individual point is a single participant’s segregation values computed from two different scan sessions. Lines represent linear regression between segregation values computed on each individual on different scan days, with 68^th^ percentile bootstrapped confidence interval shaded. The somatomotor dorsal network is excluded from these results as after removal of low-quality scans for those ROIs there are too few datapoints for a reliable indicator of robustness (see supplemental fMRI details for further discussion).

**Supplementary Figure 5**: **Diabetes is associated with spatially distinct network segregation loss beyond aging.** Networks especially affected by diabetes are cingulo-opercular, default mode, fronto-parietal, salience, and somatomotor lateral networks. Individual points represent segregation computed from each fMRI scan. Lines represent linear regression between segregation and age with 68^th^ percentile bootstrapped confidence interval shaded.

**Supplementary Figure 6**: **Male sex is associated with similarly spatially distinct network segregation loss beyond aging in the 3T dataset.** Cingulo-opercular, default mode, salience, and somatomotor lateral networks were associated with lowered network segregation in male participants in this dataset. There is a marginally increased network segregation in males in the auditory network. Individual points represent segregation computed from each fMRI scan. Lines represent linear regression between segregation and age with 68^th^ percentile bootstrapped confidence interval shaded.

**Supplementary Figure 7**: **Male sex is associated with similarly spatially distinct network segregation loss beyond aging in the 7T dataset.** Cingulo-opercular, default mode, salience, and somatomotor lateral networks were associated with lowered network segregation in male participants in this dataset. Individual points represent segregation computed from each fMRI scan. Lines represent linear regression between segregation and age with 68^th^ percentile bootstrapped confidence interval shaded.

**Supplementary Figure 8**: **APOE4+ status is associated with network segregation loss beyond aging in different networks than those affected by diabetes and male sex.** APOE4+ status is associated with loss of functional segregation in the parieto-medial network, and marginally in the auditory network as well. Individual points represent segregation computed from each fMRI scan. Lines represent linear regression between segregation and age with 68^th^ percentile bootstrapped confidence interval shaded.

**Supplementary Figure 9: Number of genes selected for GSEA analysis does not substantially alter the primary analyses.** This figure presents a reproduction of Figure 5D (**A**), which shows the results of the GSEA analyses using the 500 most significant genes. This is presented for comparison with repeated GSEA analyses at cutoffs of 400 (**B**) and 300 (**C**) most significant genes, which do not show substantially different rank ordering of the same gene families. Non-overlapping gene families at these lower levels are not illustrated. Also when interpreting note the different axes; this is done on purpose to demonstrate the loss of power in GSEA analyses as the number of genes included for comparison shrinks.

**Supplementary Figure 10. QC-FC correlation as a function of distance in the 7T dataset.** These plots are provided as a demonstration of the sufficiency of the motion correction in the fMRI preprocessing pipeline we employed. These plots employ the same technique as prior work benchmarking this approach *(22)*, and interested readers should reference this work for more details. The x-axis is the distance (in mm) between each ROI, and the y-axis is the correlation between the QC metric (framewise displacement) and the functional connectivity between the regions. The black lines show the relative density levels and the average trend with distance (r=0.006 on day 1, r=-0.017 on day 2). We demonstrate this in the 7T dataset as each participant was scanned on two separate days (using the same approach in Supplementary Figure 11), allowing for a within-dataset validation by computing the metrics separately for each day.

**Supplementary Figure 11. QC-FC correlation distributions.** As done in previous work validating the preprocessing pipeline used in this study *(22)*, we demonstrate that the distributions of QC-FC correlations are roughly normally distributed with a low absolute value median. These plots demonstrate the counts (bins) and kernel density estimate (magenta line) of the data presented in Supplementary Figure 10, with the median correlation (dashed black line) at -0.005 on day 1 and -0.018 on day 2. As in Supplementary Figure 10, we demonstrate this in the 7T dataset as each participant was scanned on two separate days, allowing for a within-dataset validation by computing the metrics separately for each day.

Supplementary References

1. C. T. Gualtieri, L. G. Johnson, Reliability and validity of a computerized neurocognitive test battery, CNS Vital Signs. *Archives of Clinical Neuropsychology* **21**, 623–643 (2006).

2. V. V. Gusel’nikova, D. E. Korzhevskiy, NeuN As a Neuronal Nuclear Antigen and Neuron Differentiation Marker. *Acta Naturae* **7**, 42–47 (2015).

3. R. Benavides-Piccione, J. DeFelipe, Distribution of neurons expressing tyrosine hydroxylase in the human cerebral cortex. *J Anat* **211**, 212–222 (2007).

4. M. H. Soltani, R. Pichardo, Z. Song, N. Sangha, F. Camacho, K. Satyamoorthy, O. P. Sangueza, V. Setaluri, Microtubule-Associated Protein 2, a Marker of Neuronal Differentiation, Induces Mitotic Defects, Inhibits Growth of Melanoma Cells, and Predicts Metastatic Potential of Cutaneous Melanoma. *Am J Pathol* **166**, 1841–1850 (2005).

5. R. A. DeGiosio, M. J. Grubisha, M. L. MacDonald, B. C. McKinney, C. J. Camacho, R. A. Sweet, More than a marker: potential pathogenic functions of MAP2. *Front. Mol. Neurosci.* **15** (2022), doi:10.3389/fnmol.2022.974890.

6. J. Middeldorp, E. M. Hol, GFAP in health and disease. *Prog Neurobiol* **93**, 421–443 (2011).

7. M. V. Sofroniew, H. V. Vinters, Astrocytes: biology and pathology. *Acta Neuropathol* **119**, 7–35 (2010).

8. F. Brozzi, C. Arcuri, I. Giambanco, R. Donato, S100B Protein Regulates Astrocyte Shape and Migration via Interaction with Src Kinase. *J Biol Chem* **284**, 8797–8811 (2009).

9. J. C. Deloulme, E. Raponi, B. J. Gentil, N. Bertacchi, A. Marks, G. Labourdette, J. Baudier, Nuclear expression of S100B in oligodendrocyte progenitor cells correlates with differentiation toward the oligodendroglial lineage and modulates oligodendrocytes maturation. *Mol Cell Neurosci* **27**, 453–465 (2004).

10. M. J. Hawrylycz, E. S. Lein, A. L. Guillozet-Bongaarts, E. H. Shen, L. Ng, J. A. Miller, L. N. van de Lagemaat, K. A. Smith, A. Ebbert, Z. L. Riley, C. Abajian, C. F. Beckmann, A. Bernard, D. Bertagnolli, A. F. Boe, P. M. Cartagena, M. M. Chakravarty, M. Chapin, J. Chong, R. A. Dalley, B. D. Daly, C. Dang, S. Datta, N. Dee, T. A. Dolbeare, V. Faber, D. Feng, D. R. Fowler, J. Goldy, B. W. Gregor, Z. Haradon, D. R. Haynor, J. G. Hohmann, S. Horvath, R. E. Howard, A. Jeromin, J. M. Jochim, M. Kinnunen, C. Lau, E. T. Lazarz, C. Lee, T. A. Lemon, L. Li, Y. Li, J. A. Morris, C. C. Overly, P. D. Parker, S. E. Parry, M. Reding, J. J. Royall, J. Schulkin, P. A. Sequeira, C. R. Slaughterbeck, S. C. Smith, A. J. Sodt, S. M. Sunkin, B. E. Swanson, M. P. Vawter, D. Williams, P. Wohnoutka, H. R. Zielke, D. H. Geschwind, P. R. Hof, S. M. Smith, C. Koch, S. G. N. Grant, A. R. Jones, An anatomically comprehensive atlas of the adult human brain transcriptome. *Nature* **489**, 391–399 (2012).

11. B. A. Seitzman, C. Gratton, S. Marek, R. V. Raut, N. U. F. Dosenbach, B. L. Schlaggar, S. E. Petersen, D. J. Greene, A set of functionally-defined brain regions with improved representation of the subcortex and cerebellum. *NeuroImage* **206**, 116290 (2020).

12. A. Arnatkevic̆iūtė, B. D. Fulcher, A. Fornito, A practical guide to linking brain-wide gene expression and neuroimaging data. *NeuroImage* **189**, 353–367 (2019).

13. J. Quackenbush, Microarray data normalization and transformation. *Nat Genet* **32 Suppl**, 496–501 (2002).

14. M. Hawrylycz, J. A. Miller, V. Menon, D. Feng, T. Dolbeare, A. L. Guillozet-Bongaarts, A. G. Jegga, B. J. Aronow, C.-K. Lee, A. Bernard, M. F. Glasser, D. L. Dierker, J. Menche, A. Szafer, F. Collman, P. Grange, K. A. Berman, S. Mihalas, Z. Yao, L. Stewart, A.-L. Barabási, J. Schulkin, J. Phillips, L. Ng, C. Dang, D. R. Haynor, A. Jones, D. C. Van Essen, C. Koch, E. Lein, Canonical genetic signatures of the adult human brain. *Nat Neurosci* **18**, 1832–1844 (2015).

15. B. B. Avants, N. J. Tustison, G. Song, P. A. Cook, A. Klein, J. C. Gee, A reproducible evaluation of ANTs similarity metric performance in brain image registration. *Neuroimage* **54**, 2033–2044 (2011).

16. G. Gryglewski, R. Seiger, G. M. James, G. M. Godbersen, A. Komorowski, J. Unterholzner, P. Michenthaler, A. Hahn, W. Wadsak, M. Mitterhauser, S. Kasper, R. Lanzenberger, Spatial analysis and high resolution mapping of the human whole-brain transcriptome for integrative analysis in neuroimaging. *Neuroimage* **176**, 259–267 (2018).

17. E. T. Rolls, C.-C. Huang, C.-P. Lin, J. Feng, M. Joliot, Automated anatomical labelling atlas 3. *Neuroimage* **206**, 116189 (2020).

18. B. D. Fulcher, M. A. Little, N. S. Jones, Highly comparative time-series analysis: the empirical structure of time series and their methods. *Journal of The Royal Society Interface* **10**, 20130048 (2013).

19. A. Subramanian, P. Tamayo, V. K. Mootha, S. Mukherjee, B. L. Ebert, M. A. Gillette, A. Paulovich, S. L. Pomeroy, T. R. Golub, E. S. Lander, J. P. Mesirov, Gene set enrichment analysis: A knowledge-based approach for interpreting genome-wide expression profiles. *Proceedings of the National Academy of Sciences* **102**, 15545–15550 (2005).

20. A. Liberzon, C. Birger, H. Thorvaldsdóttir, M. Ghandi, J. P. Mesirov, P. Tamayo, The Molecular Signatures Database (MSigDB) hallmark gene set collection. *Cell Syst* **1**, 417–425 (2015).

21. R. T. Pillich, J. Chen, C. Churas, S. Liu, K. Ono, D. Otasek, D. Pratt, NDEx: Accessing Network Models and Streamlining Network Biology Workflows. *Current Protocols* **1**, e258 (2021).

22. R. Ciric, D. H. Wolf, J. D. Power, D. R. Roalf, G. L. Baum, K. Ruparel, R. T. Shinohara, M. A. Elliott, S. B. Eickhoff, C. Davatzikos, R. C. Gur, R. E. Gur, D. S. Bassett, T. D. Satterthwaite, Benchmarking of participant-level confound regression strategies for the control of motion artifact in studies of functional connectivity. *Neuroimage* **154**, 174–187 (2017).
