# Supplementary material for: Spatially convergent functional MRI signatures of diabetes and male sex identify genetic vulnerabilities to accelerated brain ageing
Source: Brain Commun. 2026 Sep 19;8(5):fcag347. doi: 10.1093/braincomms/fcag347 (PMC13599025; doi:10.1093/braincomms/fcag347)
Supplement: Supplementary file 1 [file fcag347_supplementary_data.zip › Supplementary_Table_Legends.pdf]

Supplementary Tables

These are the captions associated with each of the supplemental tables. As they are all substantially larger than a standard page, they are included as separate Excel files for ease of viewing. The associated file names are given at the end of each captions.

Supplementary Table 1: Associations of age effects with network segregation in the 7T dataset, corrected for repeated scans of the same individuals. File: SupplementaryTable1.xlsx.

Supplementary Table 2: Associations of diabetes effects with network segregation in the 3T dataset, with age included in the same OLS model. File: SupplementaryTable2.xlsx.

Supplementary Table 3: Associations of sex effects with network segregation in both 3T and 7T datasets, with age included in the same OLS models. File: SupplementaryTable3.xlsx.

Supplementary Table 4: Associations of diabetes and sex with network segregation in the 3T dataset, probing both individual effects and a diabetes*sex interaction effect, with age included in the same OLS model. File: SupplementaryTable4.xlsx.

Supplementary Table 5: Associations of fasting blood glucose, HbA1c and sex with network segregation in both the 3T and 7T datasets, probing both individual effects and combined glucose*sex or HbA1c*sex interaction effects, with age included in the same OLS models. File: SupplementaryTable5.xlsx.

Supplementary Table 6: Associations of APOE4 effects with network segregation in the 3T dataset, with age included in the same OLS model. File: SupplementaryTable6.xlsx.

Supplementary Table 7: Complete list of genes selected for the comparisons shown in Fig. 5A and their respective functional families. File: SupplementaryTable7.xlsx.

Supplementary Table 8: Entire GSEA results for 500 genes most significantly overexpressed in the diabetes and sex-related functional networks, showing the 10 gene sets that had the most genes overlapping with the top 500. File: SupplementaryTable8.tsv.

Supplementary Table 9: Full list of all relative expression ratios and p-values (both FDR-corrected as shown in Fig. 4B-C and uncorrected for reference) for all 14,609 genes analyzed. File: SupplementaryTable9.xlsx.
